# Machine learning-driven geochemical fingerprinting and risk characterization of mineral dust across different operational settings in El-Gedida Iron Mine, Egypt

**DOI:** 10.1007/s10653-025-02850-w

**Published:** 2025-11-17

**Authors:** Mouataz T. Mostafa, Ahmed Abdelaal, Madiha S M Osman, Hassan I. Farhat, Mariam Y. Zakaria, Reham Y. Abu Elwafa, Sahar M. Abd El-Bakey

**Affiliations:** 1 Geology Department, Faculty of Science, Arish University, Arish, North Sinai 45511 Egypt; 2 Environmental Sciences Department, Faculty of Science, Port Said University, Port Said, 42522 Egypt; 3 Geology Department, Faculty of Science, Elmergib University, Al-Khums, 40770 Libya; 4 Geology Department, Faculty of Science, Suez University, Suez, 41518 Egypt; 5 Department of Geological and Biological Sciences, Faculty of Education, Ain Shams University, Cairo, 11341 Egypt; 6 Geology Department, Faculty of Science, Sohag University, Sohag, 82524 Egypt

**Keywords:** Bahariya Oasis, Hazardous metals, Supervised classification models, Multivariate statistical analysis, Human risk assessment

## Abstract

Investigating mineral dust emitted from mining activities enables the assessment of environmental risks posed by potentially toxic elements (PTEs) and the discrimination of geochemical fingerprints characteristic of distinct operational settings. Accordingly, this study employed site-specific dust sampling, geochemical analysis of PTEs using ICP-AES, supervised machine learning (e.g., Support Vector Machine and Multinomial Logistic Regression), multivariate statistics (e.g., Principal Component Analysis), pollution and ecological indices (e.g., Pollution Load Index), and health risk modeling to delineate PTE contamination patterns, determine high-risk microenvironments, and identify geochemical fingerprints (e.g., ore-handling zones vs. confined cabins) within El-Gedida Iron Mine (Western Desert, Egypt), thereby establishing dust-borne elemental profiles as tracers for evidence-based environmental intervention. Mean PTE concentrations decreased in the order of Fe > Mn > Zn > Cr > Pb > Cu > Ni, with Cu showing extreme variability (CV = 142.6%) and a 40-fold range, linked to a localized enrichment. Composite indices exhibited substantial contamination across all samples, with a mean PLI of 2.21. Cr and Ni posed unacceptable lifetime cancer risks in children (TCR = 6.87E−04 and 2.28E−04, respectively), while Cr exhibited the highest non-carcinogenic risk (HI = 0.522), though below the critical threshold (HI < 1). Supervised machine learning models demonstrated reliable group separability and probabilistic discrimination driven by key elemental predictors (e.g., Cu), effectively extracting latent geochemical signatures, with prominent examples including the Cu–Pb-enriched fingerprint indicative of confined drilling cabins, reflecting localized accumulation from internal vehicular emissions, and the Fe–Mn lithogenic-derived signature characteristic of ore-handling zones. The Multinomial Logistic Regression (MLR) model achieved a predictive accuracy of 95.8%, highlighting the framework’s strong practical applicability.

**Supplementary Information:**

The online version contains supplementary material available at 10.1007/s10653-025-02850-w.

## Introduction

Mineral dust (MD) comprises a heterogeneous assemblage of fine particulate matter (< 63 µm) originating from natural sources (e.g., wind-driven soil erosion) and anthropogenic activities (e.g., overburden removal) (Liang et al., 2024; Noble et al., 2017; Ohata et al., 2025). Once released into the atmosphere, these particles exhibit variable residence times and undergo complex physicochemical transformations influenced by size, density, and atmospheric turbulence (Schepanski, 2018). Their environmental fate is governed by dynamic processes, including gravitational settling, turbulent dispersion, agglomeration, re-entrainment, and removal by dry or wet deposition mechanisms (Petavratzi et al., 2005). Airborne MD poses substantial threats to public health, environmental quality, and ecosystem integrity (Kasongo et al., 2024), primarily controlled by the mineralogical and chemical composition, as well as the duration of exposure (Baluchová et al., 2019; Mostafa et al., 2023). Chronic exposure to respirable particles has been linked to many severe respiratory diseases (e.g., silicosis and asbestosis), particularly in populations residing in dust-prone environments (e.g., construction and demolition zones) (Ma et al., 2020; Schlünssen et al., 2023). Following deposition, MD can reduce soil fertility, induce vegetation loss, and disrupt microbial and nutrient cycling (IbrahimPour et al., 2021). Another major concern arises from the mobilization of potentially toxic elements (PTEs) embedded in MD particles (e.g., Cd, As, and Cr), which poses significant risks due to their toxicity, bioavailability, and environmental persistence (Ekoa Bessa et al., 2021; Mazurek et al., 2016). Recently, supervised machine-learning algorithms have demonstrated high efficiency and reliability in PTE geochemical and environmental studies, owing to their ability to model the interactions among many predictors (Binetti et al., 2024; Itano & Sawada, 2024; Prasianakis et al., 2025). For instance, the Support Vector Machine (SVM) model effectively captures non-linear relationships in complex geochemical feature spaces (Sonmez et al., 2024), while Decision Tree algorithms establish easily interpretable, threshold-based rules that enhance the interpretability of environmental classifications (Dash et al., 2024).

Mismanagement of mining operations, expressed through unsustainable traditional extraction methods, inadequate waste disposal strategies, inefficient tailings containment, and the absence of effective dust suppression measures, compound the long-term environmental burden associated with MD emissions in iron ore mining areas (Abdel Maksoud et al., 2025; Niu et al., 2021; Quadros et al., 2016). Such inadequacies foster the continuous generation and surface accumulation of PTE-laden dust, which intensifies in arid environments where limited biological resilience impedes natural attenuation (Duarte et al., 2022). Notably, the acidic nature of iron mine tailings exacerbates PTE mobilization via oxidative dissolution and precipitation-induced leaching (Li et al., 2021; Nouri & Haddioui, 2016). Fine-grained mine tailings are often entrained as airborne MD, exhibiting sustained atmospheric suspension and undergoing long-distance aeolian dispersion and secondary redeposition (Kossoff et al., 2014; Young et al., 2021). This not only degrades surrounding ecosystems but also impairs mining efficiency through reduced visibility and causes equipment malfunctions during operations (Wang et al., 2023). The ecological consequences of these emissions in iron ore mining areas have been increasingly documented in recent literature (e.g., Bissang et al., 2024; Sarathchandra et al., 2022; Zeng et al., 2024). For instance, Li et al. (2021) documented severe PTE-induced infertility and reduction in bacterial richness and ecological diversity in soils surrounding an iron tailings pond in Northwest China, while Turner (2013) reported that dust fallout in Jack Hills Iron Mine (Western Australia) reduced plant physiological activity by over 50% within a 2-km radius, emphasizing the role of geochemical environmental assessments in iron mining zones as pillars for implementing remediation interventions.

Each mining activity generates a distinct dust profile influenced by its operational dynamics, exhibiting specific composition, particle size distribution, dispersion dynamics, and thereby environmental persistence, such as fugitive dust from vehicular movement on mine haul roads, coarse particulate emissions from blasting and crushing processes, and diffuse nuisance dust arising from land clearing and unsealed surfaces, especially under strong wind regimes that facilitate wider dispersion of contaminants (Duarte et al., 2022; Faber et al., 2015; Lottermoser, 2017; Petavratzi et al., 2005; Soltani et al., 2021). Operational heterogeneity also controls the mechanisms through which particles are released and transported, including air flushing during drilling, blast-induced turbulence, tire-generated resuspension, thermal uplift from engine exhausts, convective airflow disturbances caused by cooling systems, and aeolian entrainment from unconsolidated stockpiles (Sastry et al., 2015). Consequently, MD should not be regarded as a uniform emission, as the associated risks vary considerably across operational contexts, underscoring the need for site-specific characterization and tailored mitigation strategies. Although several recent studies have employed geochemical characterization approaches (e.g., Berryman et al., 2024), multivariate statistical tools (e.g., Xia et al., 2025), risk assessment indices (e.g., Kowalska et al., 2018; Sultana et al., 2023), and machine learning models (e.g., Yang et al., 2024) to evaluate MD contamination, systematic investigations linking mining operations to the corresponding dust geochemical signatures have been largely overlooked.

Bahariya Depression hosts the only commercially exploitable oolitic ironstone reserves across North Africa and Southern Europe (Salama et al., 2012), making it a strategic source of raw materials for Egypt’s iron and steel industry (Salem, 2017). These Eocene-aged deposits, predominantly located in the northeastern part of Egypt’s Western Desert, contain an estimated 270 million metric tons of ore with an average iron content of 47.6% (Baioumy et al., 2014; Said, 2017). Intensive mining activities in this region have led to the persistent release of dust-laden mining wastes, which facilitate the widespread dispersion of contaminants (e.g., PTEs) into surrounding environmental media, including cultivated and uncultivated soils (Baghdady et al., 2018). These fine particulates are easily resuspended, transported, and deposited, contributing to ecological degradation, agro-environmental constraints, and increased human exposure risks within and beyond mining areas (Rashad & Shalaby, 2007). While the geological and economic aspects of these deposits have been extensively studied (e.g., Abd El-Wahed et al., 2025; Afify et al., 2018; El-Habaak et al., 2017; Mekkawi et al., 2021; Shaik et al., 2021), environmental investigations remain scarce, and even those have neglected the geochemical characterization of MD and its site-specific ecological and health risks associated with mining activities. Accordingly, this study was designed to: (a) quantify PTE concentrations and characterize their enrichment patterns in MD samples across distinct mining operation zones (e.g., drilling sites, crushing, and unloading points) and functional environmental surfaces (e.g., truck exteriors); (b) evaluate individual and cumulative contamination levels to determine the extent of mining-induced pollution; (c) estimate site-functional ecological risks and model non-carcinogenic and carcinogenic health risks for workers and surrounding communities; (d) analyze inter-element relationships and distinguish lithogenic contributions from anthropogenic inputs through multivariate statistical techniques; and (e) improve dust classification accuracy across heterogeneous sampling categories, validate the discriminatory capacity of PTE fingerprints, and extract key geochemical tracers via supervised machine learning, thereby uncovering latent patterns linked to distinct mining operations. Ultimately, this study provides a scientific basis for assessing mineral dust and implementing targeted mitigation strategies, underpinned by the geochemical signature unique to a specific mining operation, rather than relying on mine-wide generalizations.

## Geologic setting

**Fig. 1:**
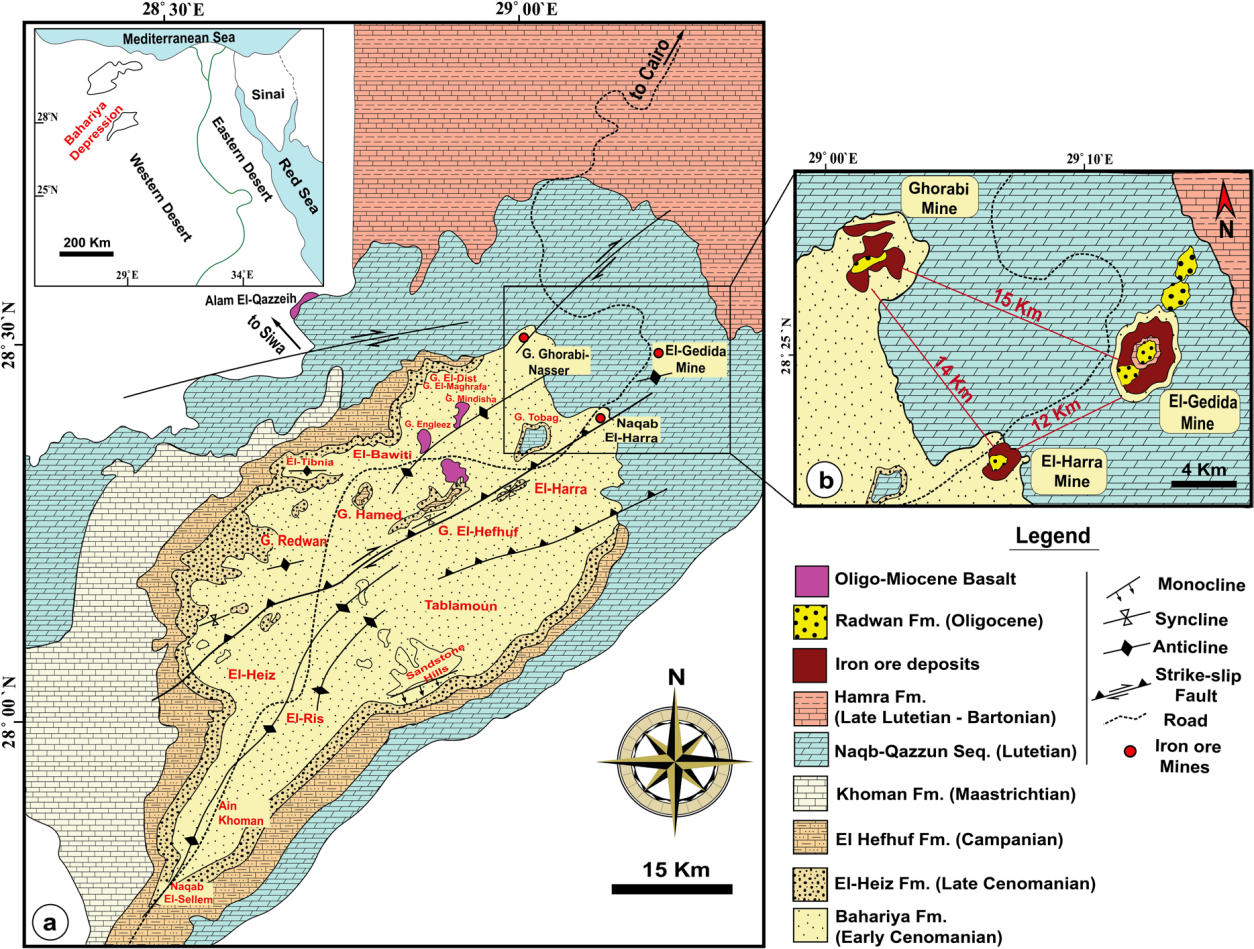
**a** Regional geological map of Bahariya Depression, Western Desert, Egypt, illustrating main lithostratigraphic units and structural features. **b** Inset map showing the distribution of iron ore mines, including the study area at El-Gedida mine (modified after Abd El-Wahed et al., 2022; Catuneanu et al., 2006; Salama et al., 2012; and Sehim, 1993)

**Fig. 2:**
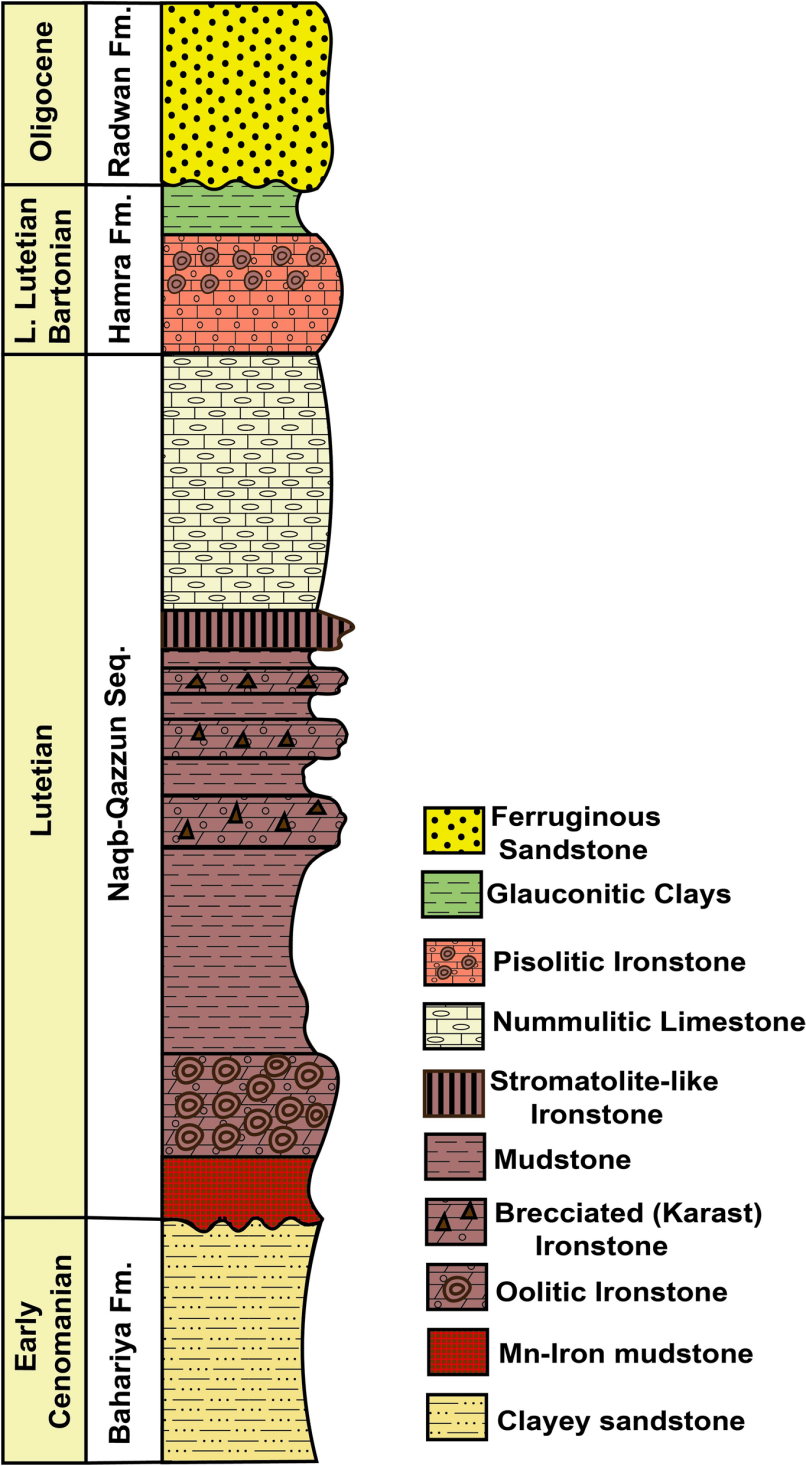
Stratigraphic column of El-Gedida mine area, modified after Baioumy et al. (2013) and Afify et al. (2016)

**Fig. 3:**
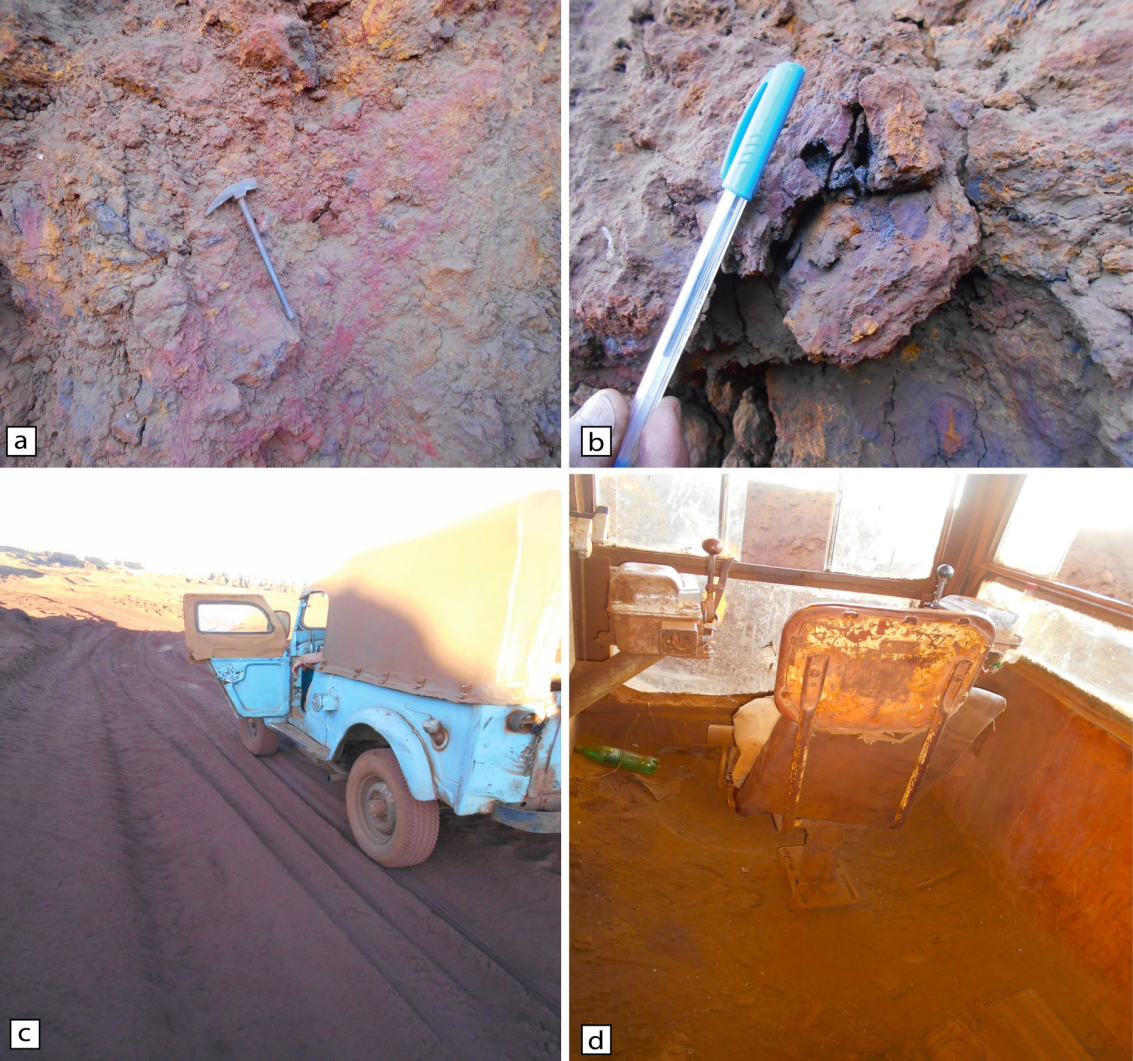
Field photographs from El-Gedida Iron Mine, Western Desert, Egypt: **a** iron ore exposure from Naqb Formation; **b** manganese oxide mineralization occurring in association with iron oxides; **c** dust accumulation on the exterior of a deteriorated field vehicle used for site transportation within the mine; and **d** heavily dust-covered interior of a field vehicle

Bahariya Depression, located 370 km southwest of Cairo, is geographically bounded between latitudes 27° 48′–28° 30′  N and longitudes 28° 32′–29° 10′ E (Fig. 1a). The area lies within a hyper-arid zone, marked by minimal precipitation and intense evaporation (Sharaky & Abdoun, 2020). Iron ore deposits are distributed over an area of approximately 11.7 km^2^ and concentrated in three main localities: El-Gedida (~ 15 km^2^), which is the focus of this study, as well as Ghorabi (~ 3.5 km^2^) and El-Harra (~ 2.9 km^2^) (Baioumy et al., 2014) (Fig. 1b). El-Gedida Iron Mine is situated within an oval-shaped depression marked by a central highland (up to 254 m a.s.l.) surrounded by lower wadi plains (~ 198 m a.s.l), and is structurally associated with an anticline fold and aligned along a dominant NE–SW fault system (Mousa et al., 2009). Main deposits are hosted in the degraded cone hills of the Lutetian Naqb–Qazzun Sequence (Fig. 2), where they occur as a laterally extensive, massive bed exhibiting red to dark reddish coloration (Fig. 3a). The ore body attains a maximum thickness of up to 35 m within the eastern and western wadis, while thinning to about 11 m across the central highland, with an overall average thickness of ~ 7.9 m (Baioumy, 2013; Hassan & Baioumy, 2007; Mousa et al., 2009). Stratigraphically, the ore-bearing horizon resides in the lower part of Naqb Formation, which consists of dolomitic and crystalline limestone interbedded with marl (Baghdady et al., 2018; Elbassyony, 2005). This formation unconformably overlies the Early Cenomanian Bahariya Formation, an older siliciclastic unit comprising glauconitic sandstones, clayey sandstones, mudstones, and uneconomic stratiform ironstone and iron sulfide bands (Baioumy & Boulis, 2012; Salama et al., 2012). The overlying Qazzun Formation consists of lagoonal nummulitic limestone, often chalky, siliceous, or dolomitic, and distinguished by melon-shaped concretions and calcite pockets (Yehia et al., 2017). Younger sedimentary cover includes the shoreline to algal reefal limestone of Hamra Formation (Late Lutetian), comprising glauconitic sediments with lateritic ironstone interbeds, skeletal remains, and intraformational conglomerates (Yehia et al., 2017) and the ferruginous quartzitic sandstones and sandy clays of the Oligocene Radwan Formation (Salem, 2017). Ore genesis has been interpreted through a range of proposed processes, including hydrothermal–metasomatic alteration, biogenic mobilization in freshwater environments, oxidative weathering of glauconitic sediments, and epigenetic enrichment (supergene and hypogene), with some evidence pointing toward karst-related precipitation and mixed hydrogenous–hydrothermal contributions (Awad et al., 2019; Baioumy, 2013; Baioumy et al., 2014; Dabous, 2002; El Shazly, 1962; El-Sharkawi, 1977; Nakhla, 1961). Mineralogically, the ore is predominantly composed of hematite, occurring in oolitic to pisolitic textures and typically present as irregular patches, pellets, and idiomorphic crystals (Afify et al., 2015; Baghdady et al., 2018). Associated mineral phases include goethite, siderite, jarosite, and pyrite, in addition to manganese oxides (e.g., pyrolusite and manganite; Fig. 3b), and minor gangue minerals such as detrital quartz, barite, glauconite, gibbsite, and clay minerals, including smectite, kaolinite, illite, and halloysite (Baghdady et al., 2018; Baioumy et al., 2014; El-Habaak, 2016; El-Habaak et al., 2017).

## Materials and methods

### Sampling approach

**Fig. 4:**
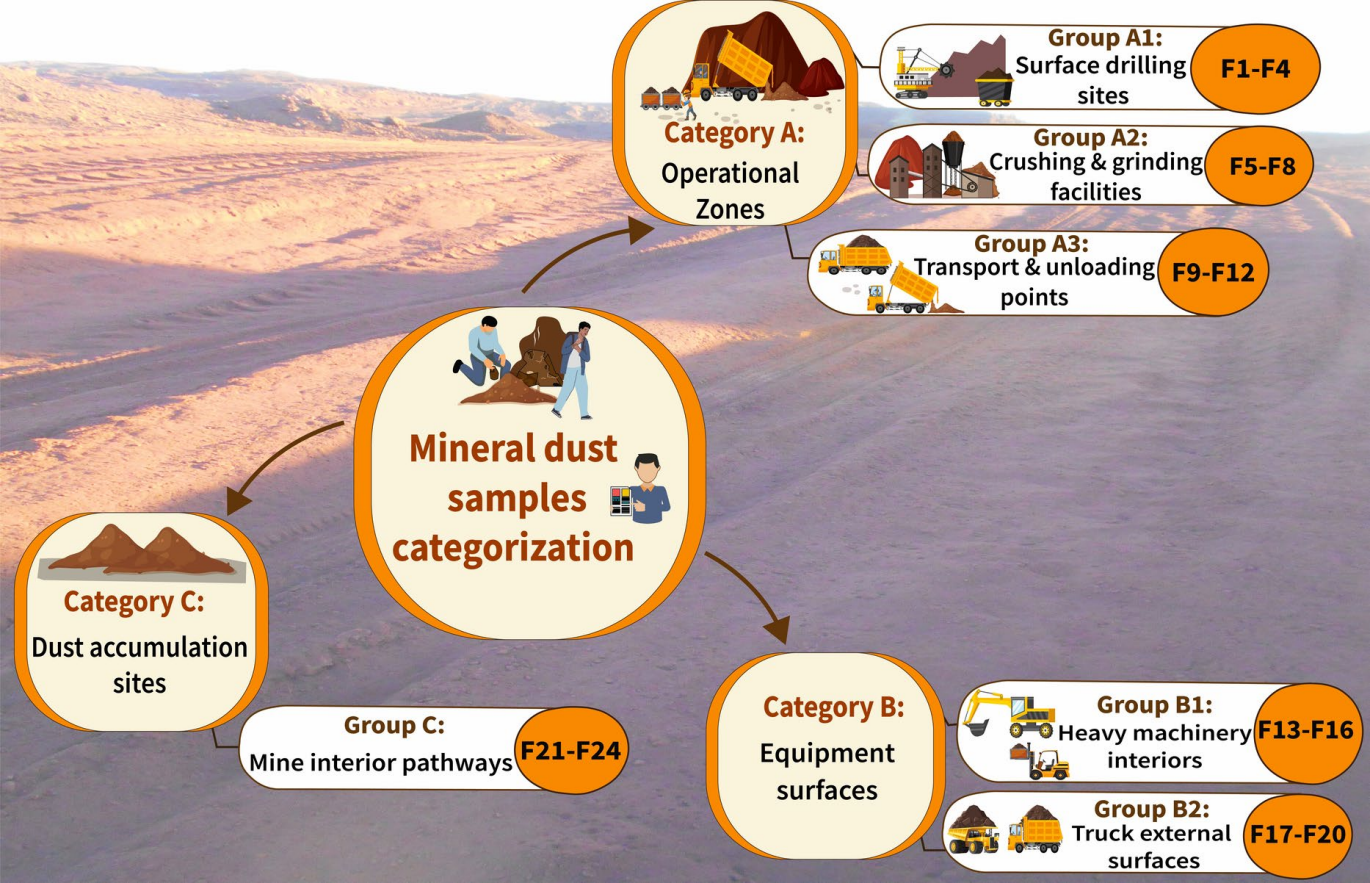
Schematic overview of mineral dust sample categorization (F1–F24) within El-Gedida Iron Mine, Western Desert, Egypt

A structured sampling strategy was developed to ensure a representative assessment that captures site-specific operational contexts and the characteristics of functional environmental surfaces. Accordingly, twenty-four fallout MD samples (F1–F24) were collected from three distinct categories: (A) operational zones, (B) equipment surfaces, and (C) dust accumulation sites on pathways, each representing key domains of dust generation, deposition dynamics, and occupational exposure risks (Figs. 3c–d and 4; Table S1). The operational zones category comprised direct dust emission sources, including surface drilling sites (F1–F4; Group A1), where mechanical fragmentation of ore-bearing strata generates substantial airborne particulates; crushing and grinding facilities (F5–F8; Group A2), where high-energy pulverization releases fine MD; and transport and unloading points (F9–F12; Group A3), where ore haulage operations and vehicular activity contribute to the resuspension of fugitive MD. The equipment exposure environments (Category B) focused on particulate deposition inside and around mining machinery, including drilling cabins and heavy machinery interiors (F13–F16; Group B1) and transport truck external surfaces (F17–F20; Group B2). The third category focused on dust accumulation sites, targeting mine interior pathways (F21–F24; Group C), which serve as high-traffic corridors for workers and heavy machinery, facilitating dust mobilization, dispersion, and subsequent redeposition.

Mineral dust samples were collected using a polyethylene brush and a plastic collection pan, ensuring the removal of deposited particulates from a clean, nonporous collection surface (Soltani et al., 2021). To prevent cross-contamination, all sampling tools were pre-cleaned with deionized water between collections. Notably, sampling was conducted during the summer period (August 2024), prior to any rainfall events, under dry and hot meteorological conditions to prevent PTE leaching and surface washout. Sampling focused on the uppermost surface layer (0–20 mm) to capture freshly deposited, highly mobile particulates with immediate inhalation risks for workers. At each sampling point, five sub-samples were collected and subsequently homogenized into a single composite sample (about 150 g) to ensure representativeness. All samples were sealed in clean, self-lock polyethylene bags, labeled, and shipped to the laboratory, where they were air-dried under ambient laboratory conditions (~ 27 °C) to eliminate any residual moisture. Air-drying at ambient temperature was employed to prevent thermal alteration or volatilization of sensitive elements (Kim et al., 2025; Koopmans & Groenenberg, 2011). The dried samples were then sieved to < 63 µm, the fraction most prone to atmospheric suspension and inhalation (Zhu et al., 2021) due to its greater surface area and reactivity, which enhance the adsorption of PTEs (Jiang et al., 2018). Sieved samples were ground using an agate mortar to obtain a fine, uniformly distributed powder suitable for subsequent analyses.

### Digestion and geochemical analysis

An accurately weighed portion of 1.0 g from each homogenized MD sample was digested with a four-acid mixture (HClO₄, H₂SO₄, HCl, and HF) using an ETHOS EASY microwave system (Milestone Inc., Italy) under a three-stage program (Liu et al., 2013; Wang et al., 2020) (Table S2). The digested solution was transferred into a 25 mL volumetric flask and diluted to the mark with ultrapure water for subsequent analysis. The concentrations of Fe, Mn, Pb, Cu, Zn, Ni, and Cr were then quantified using inductively coupled plasma atomic emission spectroscopy (ICP-AES; iCAP-6500 Duo, Thermo Scientific). The limits of detection (LOD), expressed in mg/kg, were 0.0006 for Zn, 0.002 for Mn and Ni, 0.006 for Cu, 0.008 for Pb, 0.01 for Cr, and 0.02 for Fe. These elements were selected due to their high geochemical affinity for iron-rich phases, influencing their environmental behavior and potential mobility (Ekoa Bessa et al., 2021; Sababa & Ekoa Bessa, 2022; Young et al., 2021). In this regard, all laboratory analyses were carried out at the Central Laboratories of the Egyptian Desert Research Center. To maintain analytical integrity, all glassware was acid-cleaned by immersion in 20% HNO_3_ for 24 h and subsequently rinsed with distilled water to eliminate residual impurities. Triplicate sample analyses and reagent blanks were used to evaluate analytical precision and accuracy. Moreover, instrument performance was calibrated with a certified multi-element standard solution (1000 mg/L, Merck, Germany). The analytical precision of the standard solution remained within ± 5%, with recovery rates varying between 88 and 96%.

### Assessment of PTE contamination

#### Single-element pollution indices

Comparisons of total PTE concentrations with background values are insufficient for determining the actual contamination status, as they fail to capture the cumulative influence of natural and anthropogenic contributions. Thus, a range of quantitative assessment methods (commonly termed pollution indices) has been widely integrated into recent environmental geochemical studies (Choi et al., 2024; Kabir et al., 2021; Mohanraj et al., 2024). Among these indices, the Geoaccumulation Index (I_geo_), developed by Müller (1969), is widely applied for quantifying the contamination grade of individual PTEs across diverse environmental media, including surface soils, stream sediments, and mineral dust (Cipoli et al., 2024). I_geo_ is computed as the base-2 logarithm of the ratio between the PTE measured concentration (C_n_) and 1.5 times its respective geochemical baseline (B_n_) (Eq. 1), where the constant 1.5 serves as a correction factor to accommodate natural lithogenic variability and minor anthropogenic contributions (Ekoa Bessa et al., 2022). Given the lack of a local geochemical baseline and the mobile nature of MD, the present study adopted the average shale composition of the Earth's crust as reported by Turekian and Wedepohl (1961), which is commonly applied in recent environmental studies (Mostafa et al., 2024; Nazzal et al., 2013; Satapathy & Panda, 2018). Based on the calculated I_geo_ values, contamination levels are classified into seven categories, ranging from uncontaminated (I_geo_ ≤ 0) to extremely contaminated (I_geo_ > 5), as outlined in Table S3. The Contamination Factor (CF), introduced by Hakanson (1980), was also calculated to offer a complementary and more direct indicator of PTE contamination (Abdel Maksoud et al., 2025; Opolot et al., 2023). This is supported by its computational simplicity, heightened sensitivity to concentration fluctuations, and critical role in the formulation of composite pollution indices. CF is calculated as the ratio between the measured concentration of the PTE (C_sample_) and its corresponding geochemical background level (C_background_) (Eq. 2). CF-based contamination levels were interpreted into four levels (Table S3).

1 $$\left( {{\text{I}}_{{{\text{geo}}}} } \right)_{{\text{n}}} = {\text{log}}_{2} \left( {\frac{{{\text{C}}_{{\text{n}}} }}{{1.5{ } \times {\text{B}}_{{\text{n}}} }}} \right)$$

2 $${\text{CF}} = \frac{{{\text{C}}_{{{\text{sample}}}} }}{{{\text{C}}_{{{\text{background}}}} }}$$

#### Composite pollution indices

To assess overall dust contamination across the site-functional categories, three composite pollution indices—Degree of Contamination (C_deg_), Pollution Load Index (PLI), and Nemerow Pollution Index (NPI)—were applied, all derived from the calculated CF values. Each of these indices offers a distinct analytical advantage, enabling integrated insights into contamination severity and patterns as well as the relative contribution of individual elements. The C_deg_, introduced by Hakanson (1980), provides a direct cumulative measure by summing CF values for all analyzed PTEs (Eq. 3). It is distinguished by its ease of calculation and capacity to assess the total contamination load for each sample, enabling comparisons across the environmental-functional contexts (Cuput et al., 2024). Moreover, the PLI normalizes the cumulative contamination load through a geometric mean of CFs (Eq. 4) (Tomlinson et al., 1980), allowing for characterizing pollutant accumulation in mining-derived dust (Yasin & Salih, 2025). NPI further refines this evaluation by integrating the average and the maximum CF values (Eq. 5), increasing sensitivity to dominant metal contributors and reducing the risk of underestimating localized contamination hotspots (Nemerow, 1991; Nemerow & Sumitomo, 1970; Vlasov et al., 2025). Interpretation and classification categories for these indices are summarized in Table 1.

3 $${\text{C}}_{{{\text{deg}}}} = \mathop \sum \limits_{i = 1}^{{\text{n}}} {\text{CF}}$$

4 $${\text{PLI}} = { }\left( {{\text{CF}}_{1} \times {\text{CF}}_{2} \times {\text{CF}}_{3} \times \cdots { } \times {\text{CF}}_{{\text{n}}} } \right)^{{\frac{1}{{\text{n}}}}}$$

5 $${\text{NPI}} = { }\sqrt {\frac{{({\text{CF}}_{{{\text{ave}}}} )^{2} + ({\text{CF}}_{{{\text{max}}}} )^{2} }}{{\text{n}}}} \user2{ }$$

**Table 1:** Classification criteria for composite pollution indices (Abdel Maksoud et al., 2025; Hakanson, 1980; Kabir et al., 2021; Kowalska et al., 2018; Mostafa et al., 2023, 2024; Tomlinson et al., 1980)

*Degree of contamination (C*_*deg*_*) interpretation*		
Class	C_deg_ value	Contamination level
1	< 6	Low degree of contamination
2	6–12	Moderate degree of contamination
3	12–24	Considerable degree of contamination
4	≥ 24	Very high degree of contamination

Class	PLI value	Pollution status
*Pollution categories of pollution load index (PLI)*		
1	< 1	No metal pollution
2	1	PTE loads are close to the background (baseline level of pollution)
3	> 1	Pollution exists

Class	NPI value	Pollution grade
*Classification of Nemerow pollution index (NPI)*		
I	≤ 0.7	Safety domain
II	0.7–1	Precaution domain (warning limit)
III	1–2	Slightly polluted
IV	2–3	Moderately polluted
V	≥ 3	Seriously polluted (heavy pollution)

### Ecotoxicological assessment of PTE contamination

PTEs differ in their toxicity levels, biological effects, and ecological persistence, necessitating a weighted risk-based evaluation (Özşeker & Terzi, 2025; Siddique et al., 2025). This is conducted through the Individual Ecological Risk Factor (Er), which integrates the CF of each PTE with its corresponding Toxic Response Factor $\left( {{\text{T}}_{{\text{r}}}^{{\text{i}}} } \right)$ (Eq. 6), thereby integrating contamination intensity with the toxicological potency of each element (Hakanson, 1980; Su et al., 2024). To evaluate the cumulative ecological burden on the biological community, the Er values are summed to derive the Overall Ecological Risk Index (RI), which denotes the aggregated potential risk from all studied PTEs (Eq. 7) (Yuan et al., 2025). Notably, ${\text{T}}_{{\text{r}}}^{{\text{i}}}$ values, as defined by Hakanson (1980), were assigned as follows: 5 for Cu, Ni, and Pb; 2 for Cr; and 1 for Zn, while risk levels were interpreted using the classification criteria outlined in Table S4.

6 $${\text{Er}} = {\text{T}}_{{\text{r}}}^{{\text{i}}} \times {\text{CF}}^{{\text{i}}}$$

7 $${\text{RI}} = \mathop \sum \limits_{{{\text{i}} = 1}}^{{\text{n}}} {\text{Er}}$$

### Health risk modeling of PTE exposure

To assess and characterize human health risks associated with exposure to PTEs, the framework developed by the United States Environmental Protection Agency was applied (USEPA, 2004, 2005, 2011). This framework integrates contaminant sources, exposure pathways, and receptor populations within a source–pathway–receptor conceptual model, employing dose–response toxicity values (Mohanraj et al., 2024; Wang et al., 2024a, 2024b). Distinct receptor groups, including adults and children, were considered to capture age-specific susceptibility (Rahman et al., 2021). The Average Daily Dose (ADD) [mg/kg/day] for each exposure pathway (ingestion, inhalation, and dermal contact) was calculated using Eqs. (8–10). ADD values were then divided by their respective Reference Doses (RfDs) to compute the Hazard Quotient (HQ) for each pathway (Eq. 11). The resulting HQs were subsequently summed to estimate the cumulative non-carcinogenic risk, represented by the Hazard Index (HI) (Eq. 12). Pb, Cu, Zn, Ni, and Cr were evaluated for non-carcinogenic effects based on the availability of established RfD values. Exposure doses below the RfD are not expected to cause adverse health effects, with HQ and HI values < 1 indicating negligible non-carcinogenic risks, whereas values > 1 suggest a potential for adverse health effects (Men et al., 2022; USEPA, 2002, 2004).

Carcinogenic risk was evaluated by multiplying each ADD by the corresponding Cancer Slope Factor (CSF), yielding the Individual Carcinogenic Risk (CR) values (Eq. 13), which were then summed to compute the Total Carcinogenic Risk (TCR) (Eq. 14). The carcinogenic risk assessment focused on Pb, Ni, and Cr, considering their classification as human carcinogens (IARC, 2012; USEPA, 2005). CR or TCR values below 1.00E−06 are considered insignificant, values between 1.00E−06 and 1.00E−04 fall within the acceptable risk range, and those exceeding 1.00E−04 indicate a potentially unacceptable lifetime cancer risk (Cunha-Lopes et al., 2022; USEPA, 1989, 2005). A detailed list of parameter definitions, specified values, and unit specifications used in the health risk assessment calculations is provided in Tables S5 and S6.

8 $${\text{ADD}}_{{{\text{ingestion}}}} { } = { }\frac{{{\text{C}}_{{\text{n}}} { } \times {\text{ IR}}_{{{\text{ing}}}} { } \times {\text{ ED }} \times {\text{ EF }}}}{{{\text{BW }} \times {\text{ AT}}}} \times {\text{CF}}$$

9 $${\text{ADD}}_{{{\text{inhalation}}}} = \frac{{{\text{C}}_{{\text{n}}} { } \times {\text{ IR}}_{{{\text{inh}}}} { } \times {\text{ ED }} \times {\text{ EF }}}}{{{\text{BW }} \times {\text{ AT }} \times {\text{ PEF}}}}$$

10 $${\text{ADD}}_{{{\text{dermal}}}} = { }\frac{{{\text{C}}_{{\text{n}}} { } \times {\text{ SA }} \times {\text{ AF }} \times {\text{ DAF }} \times {\text{ ED }} \times {\text{ EF }}}}{{{\text{BW }} \times {\text{ AT}}}} \times {\text{CF}}$$

11 $${\text{HQ}} = \frac{{{\text{ADD}}}}{{{\text{RfD}}}}$$

12 $${\text{HI}} = {\text{ HQ}}_{{{\text{ing}}}} + {\text{ HQ}}_{{{\text{inh}}}} { } + {\text{ HQ}}_{{{\text{dermal}}}}$$

13 $${\text{CR}} = {\text{ADD}} \times {\text{CSF}}$$

14 $${\text{TCR}} = \sum {\text{CR}}_{{\text{i}}} { } = {\text{ CR}}_{{{\text{inh}}}} { } + {\text{ CR}}_{{{\text{ing}}}} { } + {\text{ CR}}_{{{\text{dermal}}}}$$

### Statistical analysis

Standard statistical analyses, including measures of central tendency (mean and median), variability (standard deviation and coefficient of variation), and distributional properties (minimum and maximum), were conducted to characterize the PTE concentrations in MD samples. To evaluate the suitability of parametric statistical testing, the Shapiro–Wilk test was applied to each PTE within each sample category to assess compliance with the normality assumption (Marin et al., 2019; Yalcin, 2020). All corresponding *p*-values exceeded 0.05, indicating that the data were normally distributed within groups, thus supporting the application of parametric methods (e.g., ANOVA) (Table S7). Levene’s test was conducted to assess the homogeneity of variances across groups (Alrabie et al., 2021). For most PTEs, including Cu, Zn, Cr, Mn, and Fe, the assumption was met (*p* > 0.05), validating the application of one-way ANOVA to compare mean concentrations between sample groups (Guo et al., 2021). Accordingly, Pb and Ni exhibited significant variance heterogeneity (*p* < 0.05); thus, Welch’s ANOVA was employed to accommodate this violation and ensure valid inference despite heteroscedasticity (Levinton et al., 2006; Liao et al., 2017; MacMillan et al., 2017) (Table 2).

**Table 2:** One-way ANOVA with homogeneity testing summarizing mean PTE concentrations in mineral dust samples (N = 24; 4 per group) across six site-functional categories (k = 6) at El-Gedida Iron Mine

PTEs	Levene’s test (*p* > 0.05)		One-way ANOVA (*p* < 0.05)		Welch’s ANOVA (*p* < 0.05)	
	Levene’s statistic	*p*-value	F-value	*p*-value	Welch F-value	Welch *p*-value
Pb	4.207	0.0104	–	–	105.09	< 0.0001
Cu	1.973	0.1317	68.11	< 0.0001	–	–
Zn	0.585	0.7113	2.46	0.0723	–	–
Ni	3.787	0.0161	–	–	9.04	0.0035
Cr	1.536	0.2285	16.75	< 0.0001	–	–
Mn	0.489	0.7801	15.48	< 0.0001	–	–
Fe	0.805	0.5611	7.37	0.0006	–	–

F-values were calculated based on between-group variances

Degrees of freedom: df = 5 (between), 18 (within) for One-way ANOVA and df = 5 (between), 8.2 (within) for Welch’s ANOVA

Pearson’s correlation analysis was performed to evaluate inter-element relationships, providing critical insights into their geochemical behavior and potential sources (Forghani et al., 2015; Obodai et al., 2022). The correlation strength was categorized based on coefficient (*r*) values, defining strong (*r* > 0.7), moderate (0.4 ≤ *r* ≤ 0.7), and weak associations (*r* < 0.4) (Mostafa et al., 2024; Shan et al., 2013). To evaluate the suitability of the data matrix for multivariate statistical analysis, Bartlett’s test of sphericity was employed to verify the presence of significant inter-variable correlations required for factor extraction (Marin et al., 2019; Najmeddin et al., 2018). The test result (χ^2^ = 176.88, df = 21, *p* < 0.0001) indicated a statistically significant deviation from the identity matrix, thereby confirming the appropriateness of the data structure. As a result, Principal Component Analysis (PCA) was employed, following Z-score standardization and based on the correlation matrix, to decipher the complex interrelationships among PTEs and to identify their possible common sources (Dat et al., 2021). It reduces the dimensionality of the dataset by transforming it into a smaller set of orthogonal (uncorrelated) variables while preserving the variance inherent in the original data (Jose & Srimuruganandam, 2020). Only significant principal components (PCs) with eigenvalues exceeding unity were considered for interpretation (Giri & Singh, 2015). Alongside PCA, Hierarchical Cluster Analysis (HCA) was employed to classify both PTEs and site- and object-specific sampling units into distinct clusters (Vesković et al., 2024). Ward’s linkage clustering with correlation distance was applied to PTEs to identify geochemical affinities and potential common origins (Patinha et al., 2015). Furthermore, samples were classified using squared Euclidean distance to differentiate sample groups based on absolute concentration differences and site-specific dust accumulation (Bourliva et al., 2017), with the results visualized in dendrograms. Notably, IBM SPSS Statistics (version 29) was utilized for normality assessment, descriptive analysis, and multivariate statistics.

### Supervised machine learning for discriminative modeling

Four supervised machine learning algorithms were applied to assess the reliability of a priori sample categorization and to characterize the geochemical signatures of MD samples. The models were used to evaluate classification accuracy, interpret feature importance, and elucidate site-functional differentiation. In particular, Multinomial Logistic Regression (MLR) served as a statistical baseline to estimate predictor influence; the Decision Tree Classifier (DTC) extracted threshold-based elemental rules; the Support Vector Machine (SVM) captured non-linear separability with confidence-based outputs; and Partial Least Squares Discriminant Analysis (PLS-DA) revealed latent geochemical structures and identified key discriminatory variables. Prior to model implementation, all elemental concentration data were preprocessed to ensure statistical comparability across predictors. PTE concentrations were log-transformed to minimize skewness and standardized to zero mean and unit variance prior to model training (Changyong et al., 2014; Liu et al., 2024). Each algorithm was validated using the appropriate cross-validation scheme (fivefold for DTC, threefold for SVM, and Leave-One-Out for PLS-DA), while MLR performance was assessed through AIC/BIC metrics, likelihood ratio tests, and confusion matrix analysis (Bibriescas & Whittaker, 2023; Lasalvia et al., 2022; Muntasir Nishat et al., 2022). All supervised models were developed using a hybrid workflow combining Python 3.11 and scikit-learn (version 1.2.2) for algorithmic implementation and OriginPro 2025b graphical interface for model training, cross-validation, and high-resolution visualization. No missing values were detected, and outliers were retained due to their site-specific geochemical significance.

#### Multinomial logistic regression model

A Multinomial Logistic Regression (MLR) model, trained using maximum likelihood estimation, was constructed to classify MD samples across operational settings by computing class membership probabilities derived from their multivariate geochemical signatures (Raaschou-Nielsen et al., 2023; Sun et al., 2022). PTE concentrations were normalized, and sample groups were encoded as six categorical response classes. Model training converged after 100 iterations, achieving a − 2 log-likelihood of 6.61, with an Akaike Information Criterion (AIC) of 86.61 and a Bayesian Information Criterion (BIC) of 133.73, indicating high model parsimony. Predictive performance was assessed through confusion matrix analysis, yielding an overall classification accuracy of 95.8%. A likelihood ratio test confirmed the statistical significance of the full model over the null model (χ^2^ = 79.40, df = 35, *p* = 0.000027). Notably, Group A2 was set as the reference category, allowing all model coefficients to indicate relative geochemical contrasts with this baseline group.

#### Decision tree classifier

The Decision Tree Classifier (DTC) algorithm was constructed to evaluate the diagnostic capacity of PTE predictors in distinguishing between the six predefined sample categories (A1–C) as a function of their geochemical profiles (Li et al., 2022; Shaziayani et al., 2022). The model was constructed using a dataset comprising 24 samples with equal representation (n = 4) for each zone and seven PTEs as input predictors. The tree induction was based on the Gini impurity index as the splitting criterion and the optimal tree structure was pruned using the 1-standard error (1-SE) rule to prevent overfitting. The tree was trained using a minimum leaf node size of four samples and validated via fivefold cross-validation.

#### Support vector machine classification

Support Vector Machine (SVM) classification was employed to explore class separability and provide probabilistic confidence for each class prediction based on PTE fingerprints as the influential predictors (Farooq et al., 2024; Salazar-Rojas et al., 2022; Wang et al., 2022). The SVM model was trained using a radial basis function (RBF) kernel due to the expected non-linear separability between sample groups, with the regularization parameter (C) set to 1, gamma configured to scale for automatic adjustment based on the input feature space, and a convergence tolerance threshold set at 0.001. One representative sample per group was used to train the model, focusing on interpretability and group distinctiveness rather than predictive generalization. The model enabled probability estimation and the shrinking heuristic was activated to optimize support vector selection. Classification was performed using a one-vs-rest (OvR) decision function. A threefold cross-validation procedure was conducted to evaluate the model’s internal consistency, yielding modest fold accuracies (0.25, 0.125, and 0.125) and an overall training accuracy of 41.67%. Nevertheless, the model’s probability scores captured meaningful geochemical divergence among groups.

#### Partial least squares discriminant analysis

To model geochemical fingerprints across operationally and environmentally distinct site categories, Partial Least Squares Discriminant Analysis (PLS-DA) was applied (Feng et al., 2022). The model was constructed by assigning the concentrations of PTEs as independent variables (X) and the sample categories as the dependent variable (Y). All variables were mean-centered and scaled to unit variance to standardize the influence of predictors and avoid the dominance of variables with large numerical ranges (e.g., Fe). Given the relatively small number of samples within this supervised modeling dataset, no external test set was defined. Instead, the entire dataset was used for both training and prediction (Groeneveld et al., 2020; Szymańska et al., 2012; Westerhuis et al., 2008). Leave-One-Out (LOO) cross-validation was employed to mitigate overfitting and determine the optimal number of latent variables (Lee et al., 2018; Rodríguez-Pérez et al., 2018), resulting in a final model retaining two components that explained 71.6% of the variance in the predictors and 65.3% in the response variable, indicating a reasonable discriminative structure. Variable Importance in Projection (VIP) scores were derived to identify the most influential geochemical markers, with a threshold of VIP ≥ 0.8 (Costa et al., 2024; Farrés et al., 2015).

## Results and discussion

### PTE concentrations and distribution patterns

The mean concentrations (mg/kg) of PTEs in the analyzed mineral dust samples followed the descending order: Fe (260,035) > Mn (22,959) > Zn (288.75) > Cr (107.17) > Pb (62.71) > Cu (46.20) > Ni (21.19) (Table 3). The elevated concentrations of Fe and Mn are mainly attributed to two interrelated factors: (a) their substantial geogenic abundance in the host ore, where Fe is predominantly hosted in hematite (the principal ore mineral) alongside goethite and siderite, and Mn occurs in oxides and hydroxides such as pyrolusite, manganite, bixbyite, and romanechite (Baghdady et al., 2018; Baioumy et al., 2013); and (b) the intensified release induced by mechanical fragmentation and ore handling operations. This is supported by their high standard deviations (SD = 36,000 for Fe; 5,501 for Mn), and statistically confirmed by one-way ANOVA, which revealed significant variability across site-functional groups (Fe: F = 7.37, *p* = 0.0006; Mn: F = 15.48, *p* < 0.0001), highlighting the operational-driven heterogeneity (Table 2). Notably, Category A, including zones of direct engagement with the ore, exhibited the highest Fe and Mn concentrations, as crushing, comminution, and loading operations in these settings enhance the mechanical breakdown of ore-bearing rocks and facilitated the release of fine, metal-rich particulates under dry and aerodynamically agitated conditions (e.g., turbulence induced by conveyor systems and vehicular movement) (Lottermoser, 2017; Soltani et al., 2021). In alignment with these findings, the mean Mn concentration exceeded the geochemical reference values (Table 3) reported by Wedepohl (1995) (527 mg/kg in the continental crust), Turekian and Wedepohl (1961) (850 mg/kg for elements distribution in shale sedimentary rocks), and Yaroshevsky (2006) (1,000 mg/kg in the Earth’s crust) by approximately 23 to 44 times, while Fe also showed about 5 to eightfold enrichment relative to its corresponding background values.

**Table 3:** PTE concentrations in mineral dust samples from different site-functional categories at El-Gedida Iron Mine, Western Desert, Egypt (mg/kg)

Samples		Pb	Cu	Zn	Ni	Cr	Mn	Fe
A) Operational zones								
A1) Surface drilling sites								
F1		73.59	8.96	285.53	18.56	97.99	20,946	275,290
F2		77.97	12.05	324.68	20.63	118.34	27,401	316,228
F3		66.88	10.66	284.88	21.46	98.64	22,623	271,653
F4		68.63	9.58	270.08	17.30	94.80	21,822	262,931
Mean		71.77	10.31	291.29	19.49	102.44	23,198	281,526
A2) Crushing & grinding facilities								
F5		79.54	8.93	294.38	18.62	100.68	23,564	295,772
F6		84.95	10.94	354.65	23.54	124.21	26,687	351,489
F7		81.16	8.21	291.52	19.87	103.16	22,612	299,793
F8		70.98	9.32	272.12	18.46	96.68	18,372	254,801
Mean		79.16	9.35	303.17	20.12	106.18	22,809	300,464
A3) Transport & unloading points								
F9		20.23	9.79	285.32	17.34	75.21	31,969	214,190
F10		26.49	5.85	314.02	17.80	75.85	30,146	265,075
F11		22.78	7.26	285.53	15.73	67.49	28,506	230,251
F12		23.29	6.43	260.59	16.23	64.42	23,923	227,743
Mean		23.20	7.33	286.37	16.78	70.74	28,636	234,315
B) Equipment interior environments & exterior surfaces								
B1) Drilling cabins & heavy machinery interiors								
F13		78.14	170.93	296.29	28.62	129.51	24,550	236,659
F14		92.55	149.69	321.30	32.81	143.19	23,373	252,336
F15		101.16	169.23	363.04	37.05	161.62	26,408	285,112
F16		55.70	234.50	282.54	23.95	114.38	29,909	234,914
Mean		81.89	181.09	315.79	30.61	137.18	26,060	252,255
B2) Transport truck external surfaces								
F17		53.62	53.58	259.42	21.65	127.81	13,362	216,780
F18		51.34	35.10	284.85	22.70	135.40	14,640	222,107
F19		56.74	73.16	236.11	20.33	119.09	12,112	211,485
F20		58.94	69.62	244.35	23.65	123.15	12,088	213,452
Mean		55.16	57.87	256.18	22.08	126.36	13,051	215,956
C) Dust accumulation sites on pathways (mine interior pathways)								
F21		64.22	12.51	291.23	18.21	103.32	25,574	288,543
F22		69.14	10.89	273.46	17.91	101.55	23,376	267,940
F23		60.71	11.43	272.88	16.73	95.03	24,795	268,322
F24		66.23	10.26	281.33	19.51	100.44	22,248	277,981
Mean		65.08	11.27	279.73	18.09	100.09	23,998	275,697
Descriptive statistical analysis								
Maximum		101.16	234.50	363.04	37.05	161.62	31,969	351,489
Minimum		20.23	5.80	236.11	15.73	64.42	12,088	211,485
Mean		62.71	46.20	288.75	21.19	107.17	22,959	260,035
Median		66.56	10.90	285.10	19.69	102.35	23,470	264,003
Standard deviation		21.70	65.90	29.94	5.21	23.75	5,501	36,000
Coefficient of variation		34.61	142.60	10.37	24.61	22.16	23.96	13.84
Geochemical reference values								
^a^Turekian and Wedepohl (1961)		20	45	95	68	90	850	47,200
^b^Wedepohl (1995)		17	14.3	52	18.6	35	527	30,890
^c^Yaroshevsky (2006)		16	47	83	58	83	1000	46,500
Comparison with iron mining sites worldwide								
GEG Mine, Iran (Soltani et al., 2021)	Mineral dust	2.1	236.80	–	99.33	–	–	380.70
Yeshan Mine, China (Young et al., 2021)	Mine tailings	27.3	1047.47	161.89	–	32.7	–	–
Ma’anshan Mine, China (Niu et al., 2021)	Mining pit soil	17.1	122.00	179.00	10.90	7.4	–	–

^a^Distribution of the elements in Earth’s Crust in sedimentary rocks (shales)

^b^Chemical composition of the Continental Crust (upper continental crust)

^c^Abundances of chemical elements in the Earth’s Crust

Zn showed consistent distribution across all environmental-functional sample groups, as evidenced by its low CV (10.37%) and statistically non-significant variability (ANOVA: F = 2.46, *p* = 0.072), suggesting a dominant geogenic origin governed by natural dispersion mechanisms (El-Alfy et al., 2019; Liu et al., 2019). Conversely, Cu demonstrated a distinctly heterogeneous distribution pattern, characterized by a wide concentration range (5.80–234.50 mg/kg), the highest coefficient of variation (CV = 142.60%), and a considerable disparity between its mean and median values, indicating notable dispersion likely driven by localized point-source emissions (Han et al., 2016; Wang et al., 2020). Dabous (2002) documented the depletion of Cu in Bahariya iron ores compared to the average crustal abundance. Accordingly, the relatively high Cu concentrations observed in Group B1 (mean = 181.09 mg/kg) are more plausibly attributed to the indirect accumulation from internal vehicular emissions, particularly from brake housing, lubricated engine components, and metallic wear (Apeagyei et al., 2011; Bourliva et al., 2017; Hini et al., 2020). ANOVA results (F = 68.11, *p* < 0.0001) confirmed the significant operational heterogeneity of Cu. Moreover, Pb, Cr, and Ni displayed a similar pattern, with their highest concentrations also recorded in Group B1. Their inter-zonal variability was statistically significant (Pb: Welch’s F = 105.09, *p* < 0.0001; Cr: F = 16.75, *p* < 0.0001; Ni: Welch’s F = 9.04, *p* = 0.0035) and their lowest means were observed in Group A3. This pattern can be explained by the contrasting particulate accumulation mechanisms between confined environments (e.g., Group B1) and open-air operational zones. For instance, Zone A3 is exposed to episodic particulate emissions and enhanced atmospheric dispersion, whereas enclosed cabins function as confined microenvironments where respirable particles progressively accumulate due to inadequate ventilation, internal abrasion, and the persistent resuspension of settled dust (Chang et al., 2023; Djeddou et al., 2025). In a broader context, the mean concentrations of Pb, Zn, Cr, and Fe in the studied MD samples exceeded those reported in previous environmental assessments conducted around iron mining sites, such as GEG Mine, Iran (Soltani et al., 2021), Yeshan Mine, China (Young et al., 2021), and Ma’anshan Mine, China (Niu et al., 2021), highlighting their overall elevated loadings (Table 3).

### Risk characterization of mineral dust

#### PTE contamination levels

**Fig. 5:**
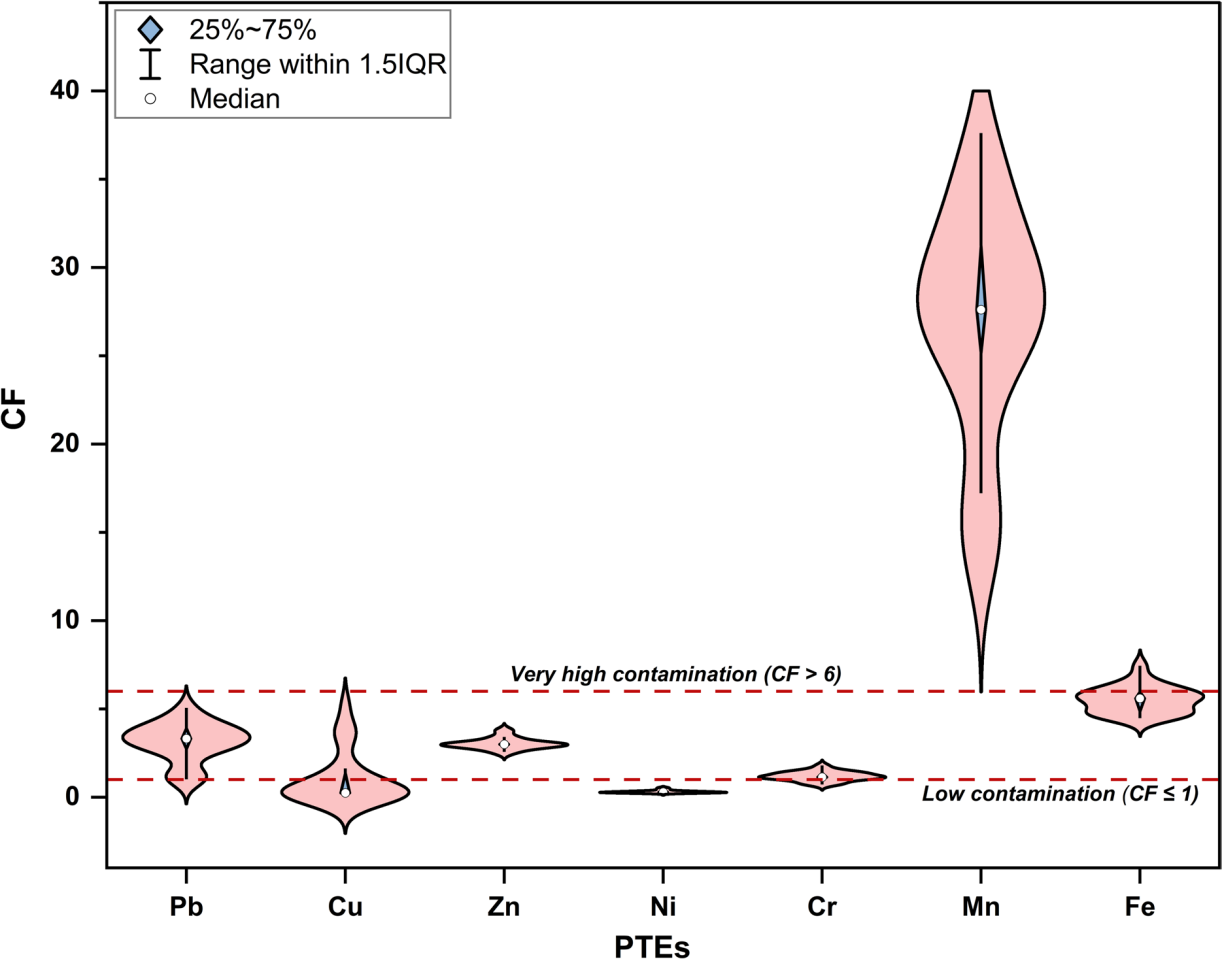
Violin plot showing the distribution and probability density of Contamination Factor (CF) values for PTEs in mineral dust samples from El-Gedida Iron Mine, Egypt

**Fig. 6:**
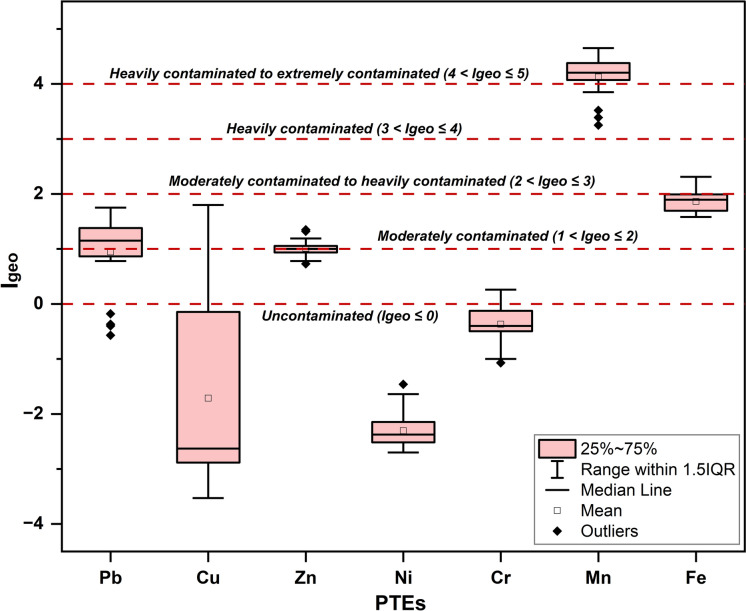
Boxplot distribution of the Geo-accumulation Index (I_geo_) values for PTEs in mineral dust samples from the El-Gedida Iron Mine, Egypt

The mean Contamination Factor (CF) values revealed a descending order as follows: Mn (27.01, very high contamination, Class 4) > Fe (5.51, considerable contamination, Class 3) > Pb (3.14, considerable contamination, Class 3) > Zn (3.04, considerable contamination, Class 3) > Cr (1.19, moderate contamination, Class 2) > Cu (1.03, moderate contamination, Class 2) > Ni (0.31, low contamination, Class 1) (Fig. 5). According to Baioumy et al. (2013), these high CF values for Mn could be attributable to its occurrence as Mn-rich cementing materials occupying the interstitial spaces between Fe-bearing minerals. Notably, Ni was consistently classified within the low contamination class (CF ≤ 1), indicating limited variability across the site’s functional groups (Table S8). Additionally, Cu displayed a localized contamination pattern, with all samples exhibiting CFs < 1 except those from Category B, especially Group B1 (F13–F16), which demonstrated considerable contamination (CF = 3.33–5.21). This anomaly implicates enclosed equipment interiors as discrete point sources of Cu accumulation. Such enrichment likely arises from multiple in-cabin emission pathways characteristic of aged mining vehicles, including abrasive wear of Cu-based components (e.g., brake pads and bearings), diesel engine emissions rich in metallic particulates, and progressive degradation of exposed electrical wiring and insulation materials in poorly ventilated compartments (Barros et al., 2019; Hulskotte et al., 2007; Straffelini et al., 2015; Terrones-Saeta et al., 2020). This interpretation is supported by comparing mean CF values between different site-functional categories, wherein Group B1 recorded the highest levels also for Pb (4.10), Zn (3.32), Cr (1.53), and Ni (0.45), representing an anthropogenic enrichment pattern driven by confined mechanical environments. In contrast, Groups A3 and B2 reported the lowest mean CFs for Pb (2.76 in B2; 1.16 in A3), indicating attenuated accumulation due to greater dispersion in their open and less intense operational settings. Furthermore, three key signatures were substantiated by the Geoaccumulation Index (I_geo_), which confirmed: the consistently extreme contamination by Mn across all environmental and operational settings (class 4–5: heavily to extremely contaminated, I_geo_ = 3.25–4.65); the uniformly minimal enrichment of Ni and Cr indicative of natural origin (Class 0: uncontaminated, I_geo_ = − 2.70 to –1.46 and − 1.07 to 0.26, respectively); and the noticeable localized accumulation of Cu, Pb, and Zn within enclosed equipment environments (Group B1), where their highest I_geo_ values corresponded to Class 2 (moderate contamination) (Fig. 6, Table S9). Baioumy et al. (2013) documented Mn concentrations reaching up to 7.66 wt.% in the high-Mn iron ores of El-Gedida Mine, with total reserves estimated at 5.3 million metric tons, justifying these elevated contamination grades as observed by CF and I_geo_.

**Fig. 7:**
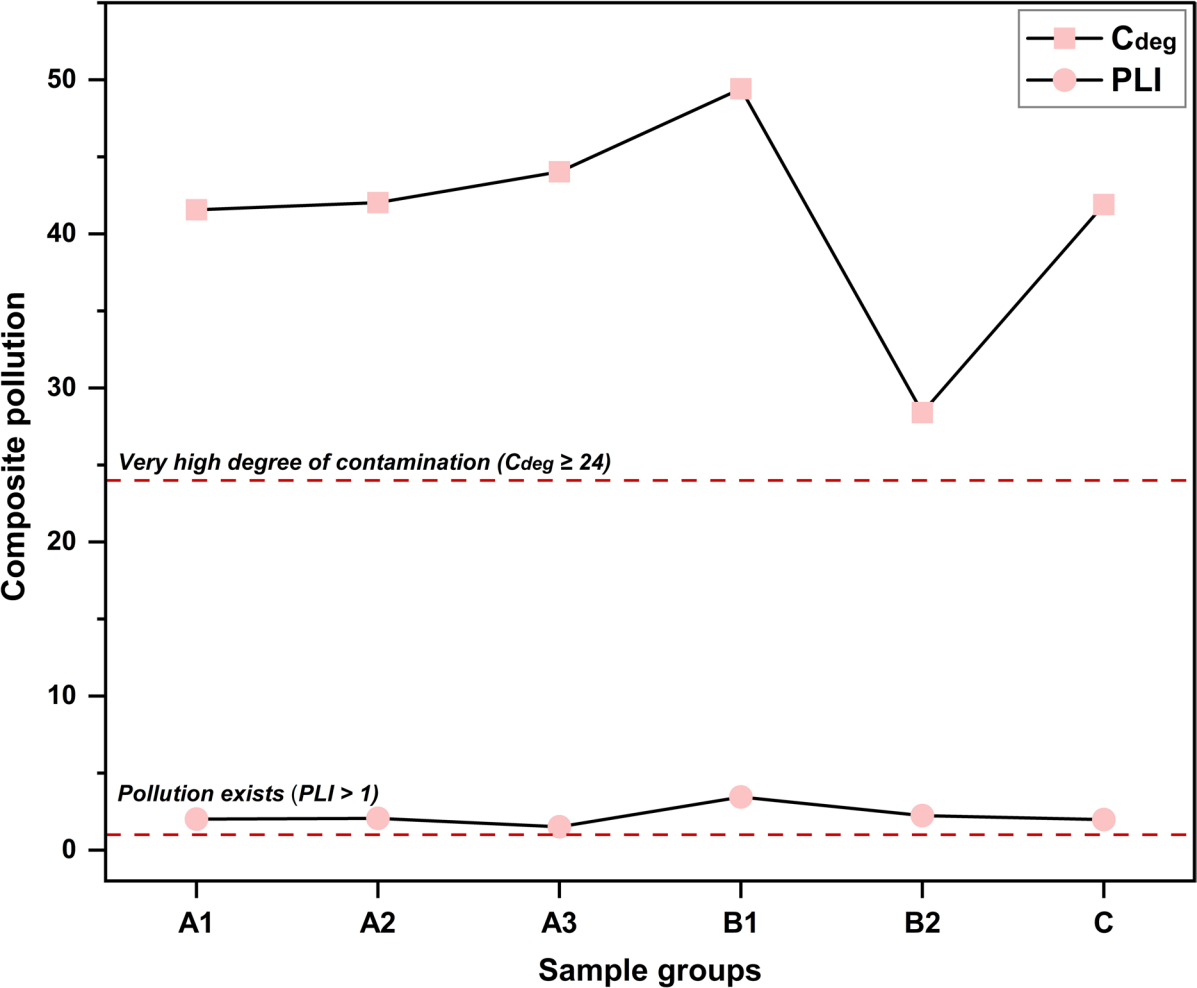
Line plot depicting the spatial variation of Degree of Contamination (C_deg_) and Pollution Load Index (PLI) across distinct sample categories in El-Gedida Iron Mine, Egypt

**Fig. 8:**
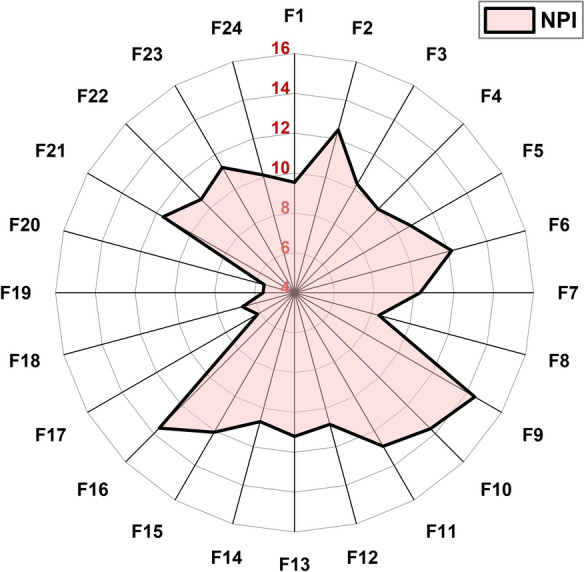
Radar plot demonstrating the spatial distribution of the Nemerow Pollution Index (NPI) across 24 dust sampling sites (F1–F24) from El-Gedida Iron Mine, Egypt

All investigated samples fell within the highest pollution category based on the three composite indices, with a mean NPI of 10.45 (≥ 3, indicating serious pollution), a mean PLI of 2.21 (> 1, confirming the presence of pollution), and a mean C_deg_ of 41.23 (≥ 24, denoting a very high degree of contamination) (Table S10). Group B1 (drilling cabins and heavy machinery interiors) exhibited the highest PLI (3.45) and C_deg_ (49.42), primarily due to the poorly ventilated nature of these microenvironments (Fig. 7). The intense particulate emissions generated by ore grinding activities were insufficiently dispersed, a condition aggravated by the deteriorated state of outdated equipment lacking modern filtration systems, contributing to the sustained retention of airborne contaminants, in contrast to the dilution effect typically observed in open, wind-exposed settings (Hudda & Fruin, 2018; Soltani et al., 2021). Conversely, Group B2 (transport truck external surfaces) recorded the lowest mean NPI (6.00) and C_deg_ (28.40), likely due to the low adhesive potential of nuisance dust originating from diffuse surface disturbance activities (Isaifan et al., 2019). This effect is compounded by the dry, hot ambient conditions of this open-pit environment, which limit capillary forces and inter-particle cohesion, reducing the potential for persistent contaminant accumulation on mobile external surfaces during haulage operations and re-suspension events when compared to stationary ground-level zones (Soltani et al., 2021). Notably, although Group A3 (F9-F12) recorded the highest NPI value (12.95), it exhibited the lowest mean PLI (1.51), highlighting the contrasting computational behavior of the indices, with NPI being highly responsive to extreme concentrations (Fig. 8), whereas PLI reflects the broader elemental loading relative to background concentrations. Nevertheless, Group A3 remained classified within the polluted category across all indices and is significantly affected by the dispersion of particulate matter arising from frequent truck movement and ore tipping during loading and unloading activities along open haul routes (Lottermoser, 2017; Wang et al., 2023).

#### Ecotoxicological and human health risks

Ecological risks associated with all PTEs remained low (Er < 40), with Mn (37.61, Group A3) and Pb (25.30, Group B1) representing the highest individual values (Table 4). Similarly, the Overall Ecological Risk Index (RI) supported this low-risk profile (RI < 150), averaging 54.81 and peaking at 85.29 in sample F15 (Group B1), thus reflecting a limited ecological burden under prevailing conditions (Table 4). These findings can be explained by the low ${\text{T}}_{{\text{r}}}^{{\text{i}}}$ values of the studied PTEs. Nevertheless, this documented low-risk status does not eliminate residual environmental concerns linked to atmospheric and hydrogeochemical transport pathways. The potential for long-range atmospheric transport of fugitive mineral dust remains significant, particularly under the region’s arid and windy climate, which promotes large-scale dispersion of particulate-bound contaminants (Sastry et al., 2015), considering that the workers’ accommodation lies about two kilometers away from the mine. Additionally, the hydrostratigraphic setting of the iron ore within the Nubian Sandstone Aquifer, a non-renewable groundwater reservoir, facilitates the leaching of elements such as Fe, Mn, and Pb into the groundwater wells, where their concentrations have been reported to exceed WHO and irrigation standards (Sharaky & Abdoun, 2020; Yehia et al., 2017). This poses a chronic environmental risk to the local communities of the Bahariya Oasis, whose inhabitants depend exclusively on this aquifer for potable water and agricultural sustenance.

**Table 4:** Ecological risk evaluation for PTEs in mineral dust from El-Gedida Iron Mine, Western Desert, Egypt

Samples	Er						RI
	Pb	Cu	Zn	Ni	Cr	Mn	
A) Operational zones							
A1) Surface drilling sites							
F1	18.40	1.00	3.01	1.35	2.18	24.64	50.58
F2	19.50	1.35	3.42	1.50	2.62	32.24	60.63
F3	16.70	1.20	3.00	1.60	2.20	26.62	51.32
F4	17.15	1.05	2.84	1.25	2.10	25.67	50.06
Mean	17.94	1.15	3.07	1.43	2.28	27.29	53.15
A2) Crushing & grinding facilities							
F5	19.90	1.00	3.10	1.35	2.24	27.72	55.31
F6	21.25	1.20	3.73	1.75	2.76	31.40	62.09
F7	20.30	0.90	3.07	1.45	2.30	26.60	54.62
F8	17.75	1.05	2.86	1.35	2.14	21.61	46.76
Mean	19.80	1.04	3.19	1.48	2.36	26.83	54.70
A3) Transport & unloading points							
F9	5.05	1.10	3.00	1.30	1.68	37.61	49.74
F10	6.60	0.65	3.31	1.30	1.68	35.47	49.01
F11	5.70	0.80	3.01	1.15	1.50	33.54	45.70
F12	5.80	0.70	2.74	1.20	1.44	28.14	40.02
Mean	5.79	0.81	3.02	1.24	1.58	33.69	46.12
B) Equipment interior environments & exterior surfaces							
B1) Drilling cabins & heavy machinery interiors							
F13	19.55	19.00	3.12	2.10	2.88	28.88	75.53
F14	23.15	16.65	3.38	2.40	3.18	27.50	76.26
F15	25.30	18.80	3.82	2.70	3.60	31.07	85.29
F16	13.95	26.05	2.97	1.75	2.54	35.19	82.45
Mean	20.49	20.13	3.32	2.24	3.05	30.66	79.88
B2) Transport truck external surfaces							
F17	13.40	5.95	2.73	1.60	2.84	15.72	42.24
F18	12.85	3.90	3.00	1.65	3.00	17.22	41.62
F19	14.20	8.15	2.49	1.50	2.64	14.25	43.23
F20	14.75	7.75	2.57	1.75	2.74	14.22	43.78
Mean	13.80	6.44	2.70	1.63	2.81	15.35	42.72
C) Dust accumulation sites on pathways (mine interior pathways)							
F21	16.05	1.40	3.07	1.35	2.30	30.09	54.26
F22	17.30	1.20	2.88	1.30	2.26	27.50	52.44
F23	15.20	1.25	2.87	1.25	2.12	29.17	51.86
F24	16.55	1.15	2.96	1.45	2.24	26.17	50.52
Mean	16.28	1.25	2.95	1.34	2.23	28.23	52.27
Statistical summary							
Maximum	25.30	26.05	3.82	2.70	3.60	37.61	85.29
Minimum	5.05	0.65	2.49	1.15	1.44	14.22	40.02
Mean	15.68	5.14	3.04	1.56	2.38	27.01	54.81
Standard deviation	5.43	7.32	0.32	0.38	0.53	6.47	12.80

Exposure assessment results (Table 5) revealed that ingestion was the dominant pathway, with children exhibiting consistently higher ADDs than adults due to lower body weight and frequent hand-to-mouth behaviors (Rahman et al., 2021). Zn showed the highest ingestion-based ADD in children (3.69E−03 mg/kg/day), followed by Cr (1.37E−03 mg/kg/day). Nonetheless, inhalation should not be overlooked in such mining contexts, as emphasized by Duarte et al. (2022), who demonstrated that drilling, crushing, and ore-handling operations entail elevated respirable dust exposure and heightened chronic respiratory risks compared with the relatively lower exposure experienced by operators in climatized settings.

**Table 5:** Health risk evaluation for PTEs in mineral dust samples from El-Gedida Iron Mine, Western Desert, Egypt via different exposure pathways

PTEs	Children ADDs			Adults ADDs		
	Ingestion	Inhalation	Dermal contact	Ingestion	Inhalation	Dermal contact
*Exposure assessment: average daily dose (ADD, [mg/(kg/day)])*						
Pb	8.02E−04	2.25E−08	2.24E−06	8.59E−05	1.26E−08	3.43E−07
Cu	5.91E−04	1.66E−08	1.65E−06	6.33E−05	9.31E−09	2.53E−07
Zn	3.69E−03	1.04E−07	1.03E−05	3.96E−04	5.82E−08	1.58E−06
Ni	2.71E−04	7.60E−09	7.59E−07	2.90E−05	4.27E−09	1.16E−07
Cr	1.37E−03	3.84E−08	3.84E−06	1.47E−04	2.16E−08	5.86E−07
*Non-carcinogenic risks: hazard quotients (HQ) and hazard indices (HI)*						

PTEs	HQ_ing_		HQ_inh_		HQ_derm_		HI	
	Children	Adults	Children	Adults	Children	Adults	Children	Adults
Pb	2.29E−01	2.45E−02	6.39E−06	3.58E−06	4.27E−03	6.53E−04	2.33E−01	2.52E−02
Cu	1.48E−02	1.58E−03	4.13E−07	2.32E−07	1.38E−04	2.11E−05	1.49E−02	1.60E−03
Zn	1.23E−02	1.32E−03	3.47E−07	1.94E−07	1.72E−04	2.63E−05	1.25E−02	1.35E−03
Ni	1.36E−02	1.45E−03	3.69E−07	2.07E−07	1.41E−04	2.15E−05	1.37E−02	1.47E−03
Cr	4.57E−01	4.90E−02	1.34E−03	7.55E−04	6.40E−02	9.77E−03	5.22E−01	5.95E−02
*Carcinogenic risks: individual (CR) and total carcinogenic risks (TCR)*								

PTEs	CR_ing_		CR_inh_		CR_derm_		TCR	
	Children	Adults	Children	Adults	Children	Adults	Children	Adults
Pb	6.82E−6	7.30E−07	1.91E−10	1.07E−10	1.90E−08	2.92E−09	6.83E−06	7.33E−07
Ni	2.28E−4	2.44E−05	6.38E−09	3.59E−09	6.38E−07	9.74E−08	2.28E−04	2.45E−05
Cr	6.85E−4	7.34E−05	1.92E−08	1.08E−08	1.92E−06	2.93E−07	6.87E−04	7.37E−05

Non-carcinogenic risk characterization showed that only Cr approached the non-carcinogenic risk threshold in children (HI = 5.22E−01), driven primarily via ingestion (HQ_ing_ = 4.57E−01) and dermal exposure (HQ_derm_ = 6.40E−02) (Table 5). Although Zn showed the highest ADD, it exhibited a negligible risk (HI = 1.25E−02), attributable to its high RfD. For adults, all HI values were well below the risk threshold (HI < 1.0), indicating negligible risk. In this context, an occupational health study at El-Gedida Mine revealed that approximately 25% of surveyed workers exhibited clinical manifestations of silicosis, linked to chronic inhalation of respirable MD and associated with inadequate personal protective equipment (PPE), limited awareness of dust-related health risks, and weak enforcement of environmental safety protocols (Mousa et al., 2014).

Carcinogenic risk assessment indicated that Cr and Ni posed the highest lifetime cancer risks for children, with TCR values of 6.87E−04 and 2.28E−04, respectively, both exceeding the acceptable threshold (1.00E−04) (Table 5). These PTEs have been epidemiologically linked to lung, gastrointestinal, renal, and nasopharyngeal cancers following chronic inhalation or ingestion exposure (Duda-Chodak & Blaszczyk, 2008; Entwistle et al., 2019; Sultan et al., 2022; Sundar et al., 2021). For adults, Cr also approached concern (TCR = 7.37E−05; within the acceptable range of 1.00E−06 to 1.00E−04). In comparison, Pb posed limited carcinogenic risk, with TCRs within the acceptable range for children (6.83E−06) and negligible for adults (7.33E−07). A recent study investigating dust dynamics in operational environments demonstrated a 116-fold increase in ultrafine particle concentrations (< 1 µm) during active working hours compared to non-operational periods (Wang et al., 2023), highlighting how intensive mining activities can elevate exposure levels and associated health risks.

### PTE associations and operation-specific source identification

**Fig. 9:**
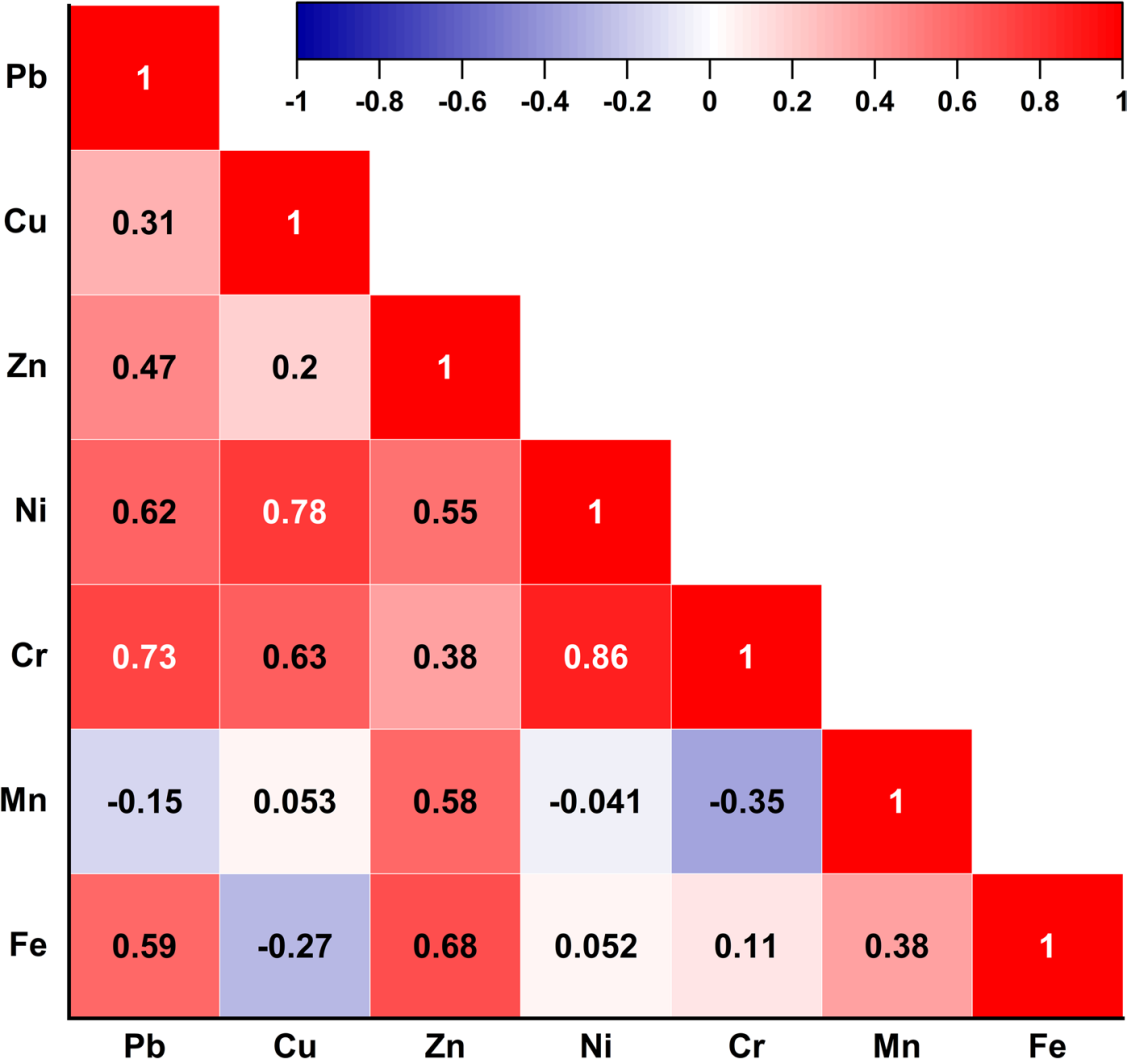
Pearson correlation heatmap showing the pairwise relationships among PTEs in dust samples from the El-Gedida Iron Mine, Egypt. Color gradients indicate the strength and direction of correlations

The dominance of Ni, Cu, and Cr in PC1 (40.85% of variance; Table 6), with respective loadings of 0.94, 0.92, and 0.84, alongside their strong to moderate inter-element Pearson correlations: Ni–Cr (*r* = 0.86), Cu–Ni (*r* = 0.78), and Cu–Cr (*r* = 0.63), pointed to confined-machinery emissions (Fig. 9). Moreover, PC2 (30.30% of variance) was defined by strong loadings of Fe (0.92) and Pb (0.79), underpinned by Fe–Pb correlation (*r* = 0.59), representing a Fe-mediated component. Fe is mechanically released during ore processing, while Pb is secondarily immobilized through sorption onto Fe(III) oxyhydroxide surfaces (Komárek et al., 2018). Considering PC3 (22.74% of variance), it was dominated by Mn (0.98), which exhibited weak to negative correlations with all other PTEs (e.g., Mn–Cr, *r* = − 0.35), demonstrating a redox-sensitive mobilization pathway driven by oxidative dissolution of Mn oxides (Sracek et al., 2021). Afify et al. (2015) identified late-stage precipitation of Mn-rich oxides (e.g., psilomelane, todorokite, and romanechite) within pores across the ore succession, reflecting redox-reactive mineral forms susceptible to remobilization under arid site conditions. Notably, Zn exhibited comparable moderate loadings in PC2 (0.63) and PC3 (0.62), indicating dual geochemical behavior, where it associated variably with Fe- and Mn-bearing oxyhydroxides through surface complexation (Dabous, 2002).

**Table 6:** Main components extracted by Principal Component Analysis (PCA) with Varimax rotation for PTEs in the studied mineral dust samples

PTEs	Principal components		
	PC1	PC2	PC3
Pb	0.49	**0.79**	− 0.21
Cu	**0.92**	− 0.22	0.13
Zn	0.35	0.63	0.62
Ni	**0.94**	0.24	0.05
Cr	**0.84**	0.38	− 0.30
Mn	− 0.09	0.07	**0.98**
Fe	− 0.17	**0.92**	0.30
*Rotation sums of squared loadings*			
Eigenvalues	2.86	2.12	1.59
Explained variance (%)	40.85	30.30	22.74
Cumulative explained variance (%)	40.85	71.14	93.88

Rotation Method: Varimax with Kaiser Normalization

Values in bold indicate strong factor loadings (≥ 0.70)

**Fig. 10:**
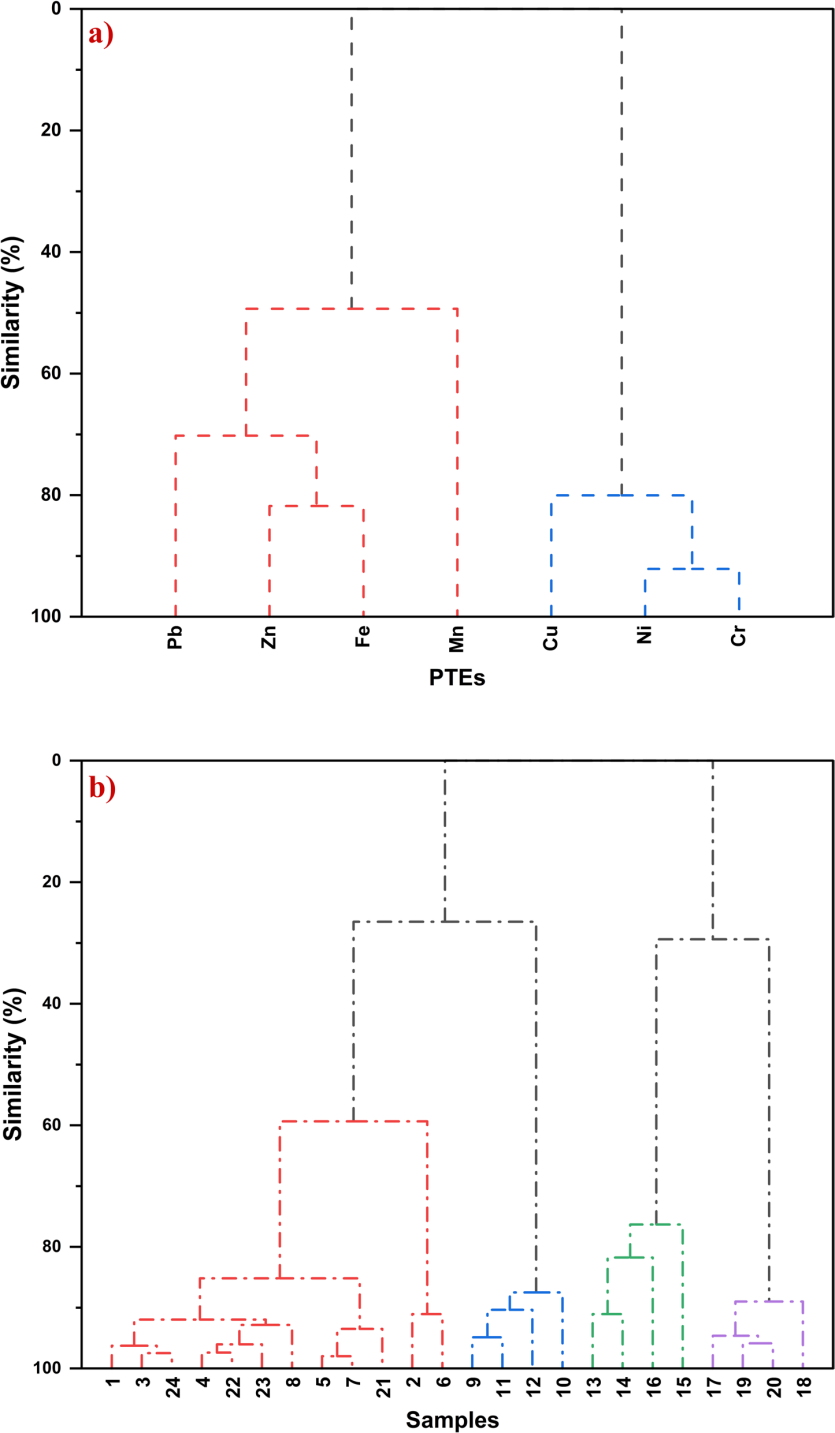
Dendrograms constructed from Hierarchical Cluster Analysis (HCA) using Ward’s Method with correlation-based Distance and Sum of Distances Linkage: **a** clustering of PTEs and **b** clustering of sampling sites from the El-Gedida Iron Mine, Egypt

HCA of PTEs (Fig. 10a) identified two distinct elemental assemblages. The first cluster (Fe, Mn, Pb, and Zn) reflected a lithogenic-associated signature, with Fe and Mn mobilized through ore disintegration during mechanical mining operations, and Pb and Zn co-clustering via surface sorption onto Fe/Mn oxyhydroxides (Krishnakumar et al., 2020). The second cluster (Cu, Ni, and Cr) indicated an equipment-derived anthropogenic origin, driven by inputs from equipment wear and fuel-derived residues (Bourliva et al., 2017; Jose & Srimuruganandam, 2020). Furthermore, sample-based HCA (Fig. 10b) revealed strong alignment with the initial functional categorization. Samples collected from heavy machinery interiors (F13–F16; Group B1) were distinctly clustered, indicating a signature linked to restricted ventilation and non-airtight enclosures allowing particulate intrusion (Jiao et al., 2025). Similarly, truck external samples (F17–F20; Group B2) clustered coherently due to shared exposure to airborne particulates and surface-bound deposition. In contrast, samples from dust-mobilizing environments (e.g., Groups A1, A2, and C) exhibited partial overlaps and demonstrated similar geochemical fingerprints. Notably, while HCA grouped Mn with Fe, likely indicating compositional co-occurrence, PCA isolated Mn, underscoring its independent redox-sensitive mobilization. This pattern suggests that Fe–Mn coupling arises from their co-occurrence within secondary oxyhydroxides formed during ore surface oxidation, where Fe acts as a structural framework stabilizing Mn phase that remain more reactive under fluctuating redox and arid oxidative conditions, influencing the retention and remobilization of trace metals (Li et al., 2023; Real et al., 2024).

### Geochemical fingerprinting of mineral dust

#### MLR-based discrimination and predictive confidence evaluation

MLR model achieved a high classification accuracy of 95.8%, correctly predicting 23 out of 24 samples. The confusion matrix (Table 7) demonstrated strong separation across all groups. Crushing facilities (Group A2), transport and unloading points (Group A3), drilling cabins (Group B1), external truck surfaces (Group B2), and mine interior pathways (Group C) each achieved 100% correct classification. For instance, the model classified sample F2 (Group A1) with 92.5% probability, sample F13 (Group B1) with near-certain classification (99.8%), and sample F21 (Group C) with 97.9%, exemplifying the distinct, confidently separable geochemical signatures characterizing these functional groups (Table S11). Only Group A1 (surface drilling sites) exhibited a single misclassification, with one sample incorrectly predicted as Group A2. The observed misclassification likely resulted from the operational similarity between drilling and crushing zones, entailing direct ore-equipment interactions and high-energy comminution. This was highlighted by the posterior probability profile, where a Group A1 sample (F1) exhibited near-equivalent assignment probabilities (54.8% to A2 and 44.1% to A1), indicating transitional geochemical signatures between these operational zones.

**Table 7:** Confusion matrix and classification performance of the Multinomial Logistic Regression (MLR) model for dust samples across site-functional categories at El-Gedida Iron Mine, Western Desert, Egypt

Observed zone (n = 4)	Predicted zone						Accuracy (%)
	A1	A2	A3	B1	B2	C	
A1	3	1	0	0	0	0	75
A2	0	4	0	0	0	0	100
A3	0	0	4	0	0	0	100
B1	0	0	0	4	0	0	100
B2	0	0	0	0	4	0	100
C	0	0	0	0	0	4	100

Overall accuracy = 95.8% (one sample misclassified out of 24)

Although no individual geochemical predictor reached statistical significance at the *p* < 0.05 threshold, consistent coefficient directions emerged across multiple groups. Pb displayed the most pronounced negative coefficients throughout the model, particularly in mine interior pathways (Group C), where β =  − 1.06, Wald = 2.85, and *p* = 0.17, suggesting discriminatory potential despite limited statistical significance. These directional trends, corroborated by odds ratios (ORs) (e.g., 0.35 for Pb in Zone C), reinforce the model’s capacity to capture latent geochemical signatures associated with functional zoning, even under moderate sample size constraints (Table S12). Conversely, Fe and Mn, while expected to vary in operational zones, showed near-zero coefficients and no statistical relevance (e.g., Fe: β =  − 0.0002, OR = 1.00 in A1; Mn: β =  + 0.0014, OR = 1.00 in A1), reflecting negligible discriminatory contribution. Interestingly, while Cu appeared strongly enriched in Group B1 samples, such an association was not retained in the regression model, likely due to multicollinearity or covariate suppression by more predictive variables. This highlights the complementary roles of descriptive (e.g., Anova) and predictive analytics (e.g., machine learning models): the former reveals patterns of association, while the latter isolates variables that drive group classification.

#### Group separability and key predictors identified by DTC

**Fig. 11:**
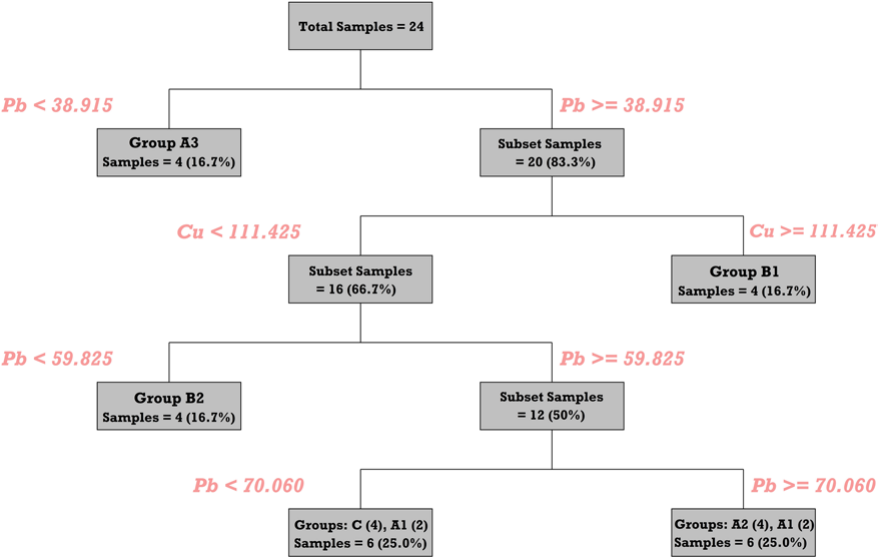
Decision tree classification of dust samples, highlighting Pb and Cu as the primary splitting variables

**Fig. 12:**
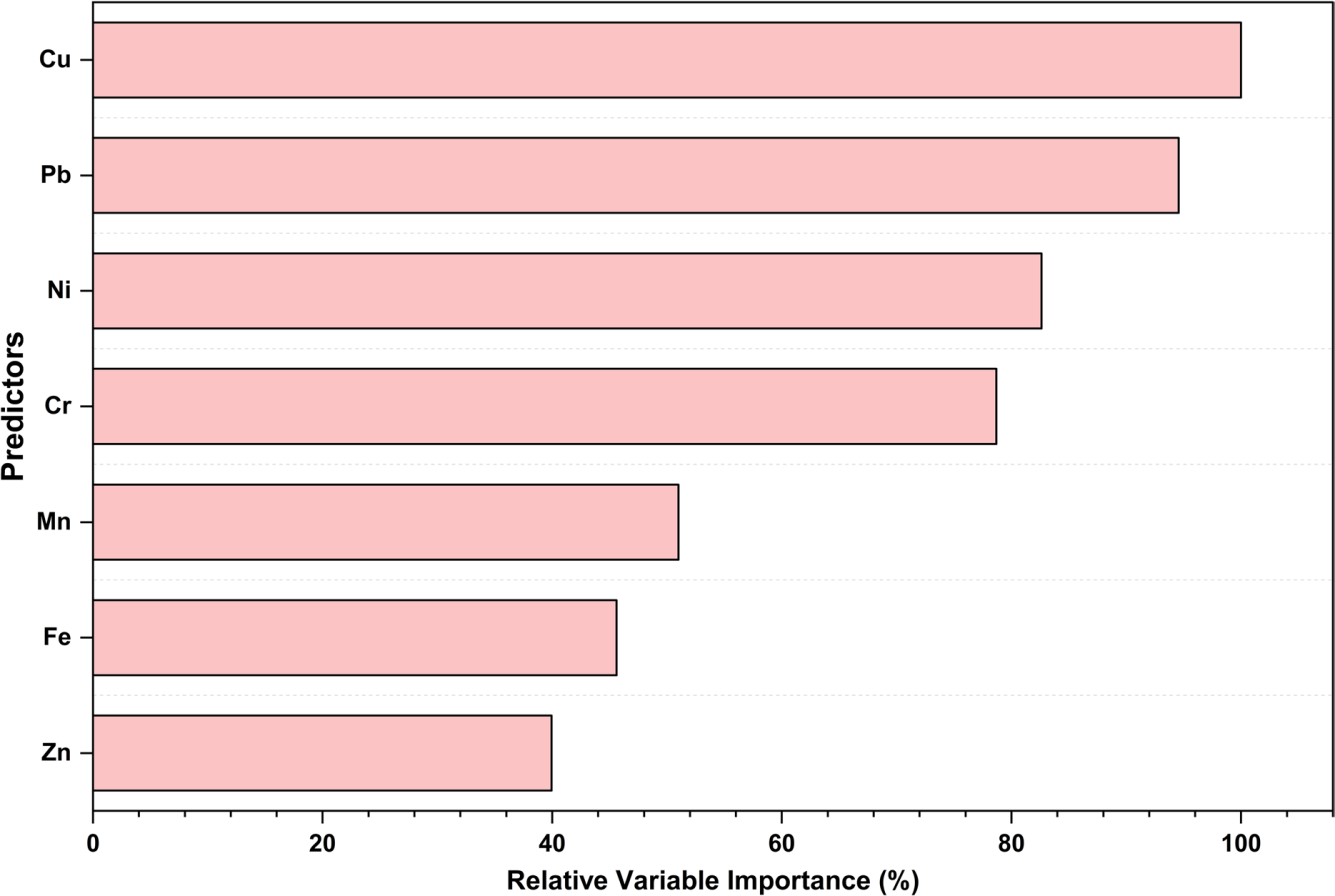
Relative importance of PTEs as classification variables identified by the Decision Tree Classifier (DTC) model

Class-specific area under the ROC curve (AUC) values demonstrated varying degrees of separability between groups. The model achieved perfect discrimination for Groups A3, B1, and B2 (AUC = 1.00), indicating the model’s great ability to distinguish these groups. In contrast, lower AUC values for Group C (0.88), Group A1 (0.80), and Group A2 (0.75) suggested partial overlap, particularly for A1, which appeared across multiple terminal nodes, notably within Groups C and A2 in the decision tree (Fig. 11), suggesting a similar mechanistic pathway of dust dispersion. Consistent with their high AUC scores, Groups A2, A3, B1, B2, and C exhibited perfect classification accuracy in the training set, contributing to an overall training accuracy of 83.3%, and reflecting the model’s robust ability to capture their geochemical signatures. In contrast, Group A1 exhibited complete misclassification, with 50% of its samples being erroneously assigned to Group A2 and the remaining 50% to Group C. On the test set, the overall accuracy decreased to 50%, primarily due to notable confusion between Groups A1, A2, and C. Notably, Group B2 maintained the highest predictive reliability with 75% accuracy (Table S13). The model yielded a relative misclassification cost of 0.05 for the training set and 0.70 for the testing set, indicating moderate generalization performance. Furthermore, assessment of predictor contributions via Relative Variable Importance (RVI) revealed that Cu (100%) and Pb (94.8%) were the most influential variables for discriminating between site-functional groups. These two elements consistently governed the major decision nodes, emphasizing their role as key tracers of anthropogenic influence (e.g., surface equipment degradation). In contrast, elements such as Mn (54.3%), Fe (46.9%), and Zn (40.1%) exhibited limited discriminative capacity (Fig. 12).

#### SVM classification and confidence-based group discrimination

**Fig. 13:**
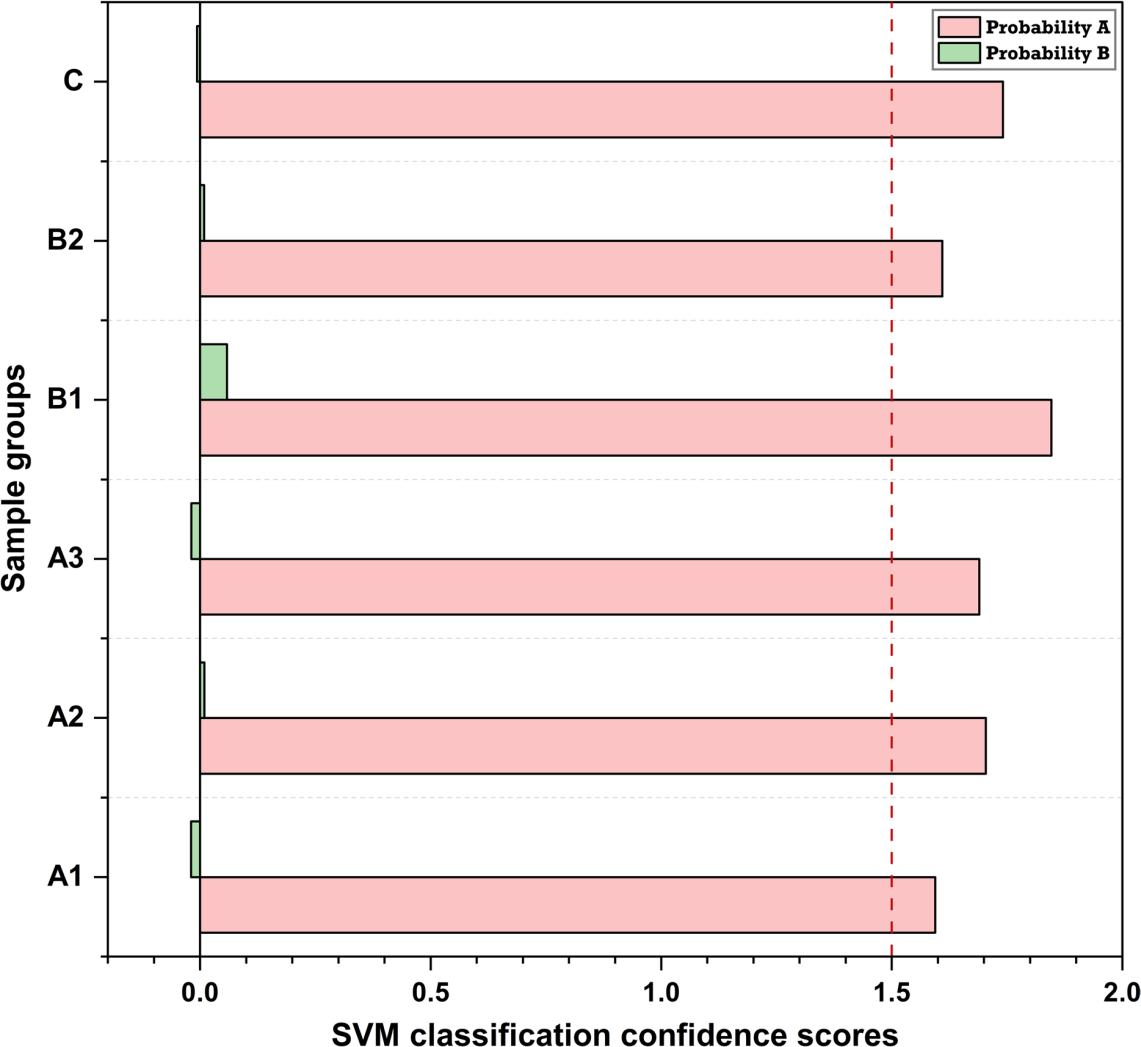
Support Vector Machine (SVM) classification confidence scores for each sample category, based on the combined probability of assignment to classes A and B. The dashed red line indicates the high-confidence threshold (Probability A > 1.5)

The highest classification confidence was achieved for Group B1 (heavy machinery interiors), with a Probability A of 1.846 and a corresponding Probability B of 0.058, reflecting a highly separable geochemical signature among the operational settings (Fig. 13). Moreover, mine interior pathways (Group C) and crushing facilities (Group A2) also exhibited relatively high classification confidence, with Probability A values of 1.741 and 1.703, respectively. In contrast, surface drilling sites (Group A1) and unloading points (Group A3) displayed lower Probability A scores, 1.594 and 1.689, respectively, accompanied by slightly negative Probability B values (− 0.0197 and − 0.0189), suggesting overlapping elemental profiles and weaker separation boundaries, which aligns with the sequence of operations of ore extraction, handling, and transfer. Notably, despite the model’s modest classification accuracy (41.67%), the probability-based outputs demonstrated well-defined confidence margins across all categories, with all Probability A scores exceeding 1.5 (Cervantes et al., 2020; Guido et al., 2024).

#### Latent geochemical signatures and outlier profiles revealed by PLS-DA

**Fig. 14:**
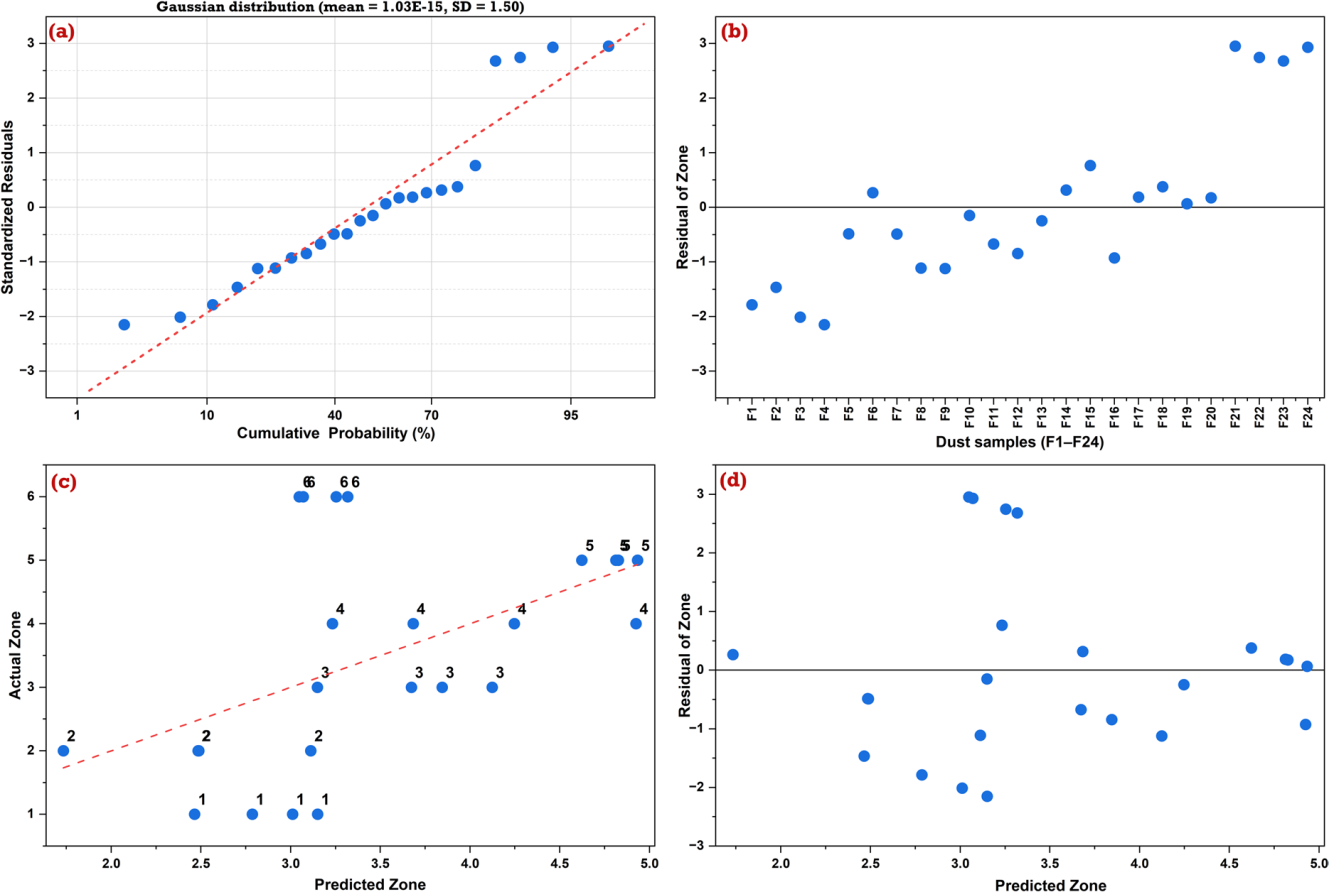
Diagnostic evaluation of the Partial Least Squares Discriminant Analysis (PLS-DA) model: **a** normal probability plot of standardized residuals, suggesting approximate normality; **b** plot of standardized residuals versus sample code (F1–F24) to assess randomness and independence; **c** observed versus predicted zone classification to evaluate model fit; and **d** residuals versus predicted values to inspect homoscedasticity and potential model bias. Zone codes range from 1 to 6, corresponding to Groups A1, A2, A3, B1, B2, and C, respectively

The diagnostic plots confirmed the statistical adequacy and classification reliability of the PLS-DA model (Fig. 14). The normal probability plot of standardized residuals (Fig. 14a) displayed an approximately Gaussian distribution (mean = 1.03E−15, SD = 1.50), supporting residual normality. Although relatively large residuals (up to ± 3) were observed in a few samples, as demonstrated in the elevated Y-space residual distances of F1, F2, and F22–F24 samples, these deviations did not indicate systematic bias, which was confirmed by the residuals versus sample code plot (Fig. 14b), revealing random local deviations with no discernible pattern. Moreover, the scatterplot of observed versus predicted zone codes (Fig. 14c) demonstrated strong alignment along the identity line, reinforcing the model's ability to extract environmentally relevant geochemical signatures with strong predictive capability across most groups. Nevertheless, misclassification was notable between Group A2 (crushing facilities) and Group A3 (unloading points), likely due to overlapping PTE signatures driven by shared dust dispersion mechanisms, atmospheric mixing, and operational interconnectivity, which could obscure distinct elemental patterns. In comparison, Group A1 (surface drilling sites) and Group B2 (external surfaces) displayed distinct classification patterns, indicating clearer geochemical separation. Residuals plotted against predicted zone codes (Fig. 14d) were randomly scattered around zero, and the predicted classifications closely aligned with observed zone codes, with most deviations falling within ± 1, indicating the absence of prediction bias toward specific classes and supporting the model’s classification reliability.

Elucidating the model’s internal structure, specific samples (e.g., F6 and F16) exhibited exceptionally high leverage values (25,288 and 31,561, respectively), indicating a disproportionate influence on the model’s latent dimensions, while F9 and F16 also showed elevated Hotelling’s T^2^ statistics (e.g., F9 = 5.81; F16 = 4.33), positioning them as geochemically distinct outliers within their assigned groups (Table S14). From an environmental geochemistry perspective, these anomalies likely reflect accumulations of specific PTEs resulting from localized activities, material inputs, or microenvironmental settings within the mine. Their identification highlights the potential of PLS-DA to detect mixed-origin dust signatures.

**Fig. 15:**
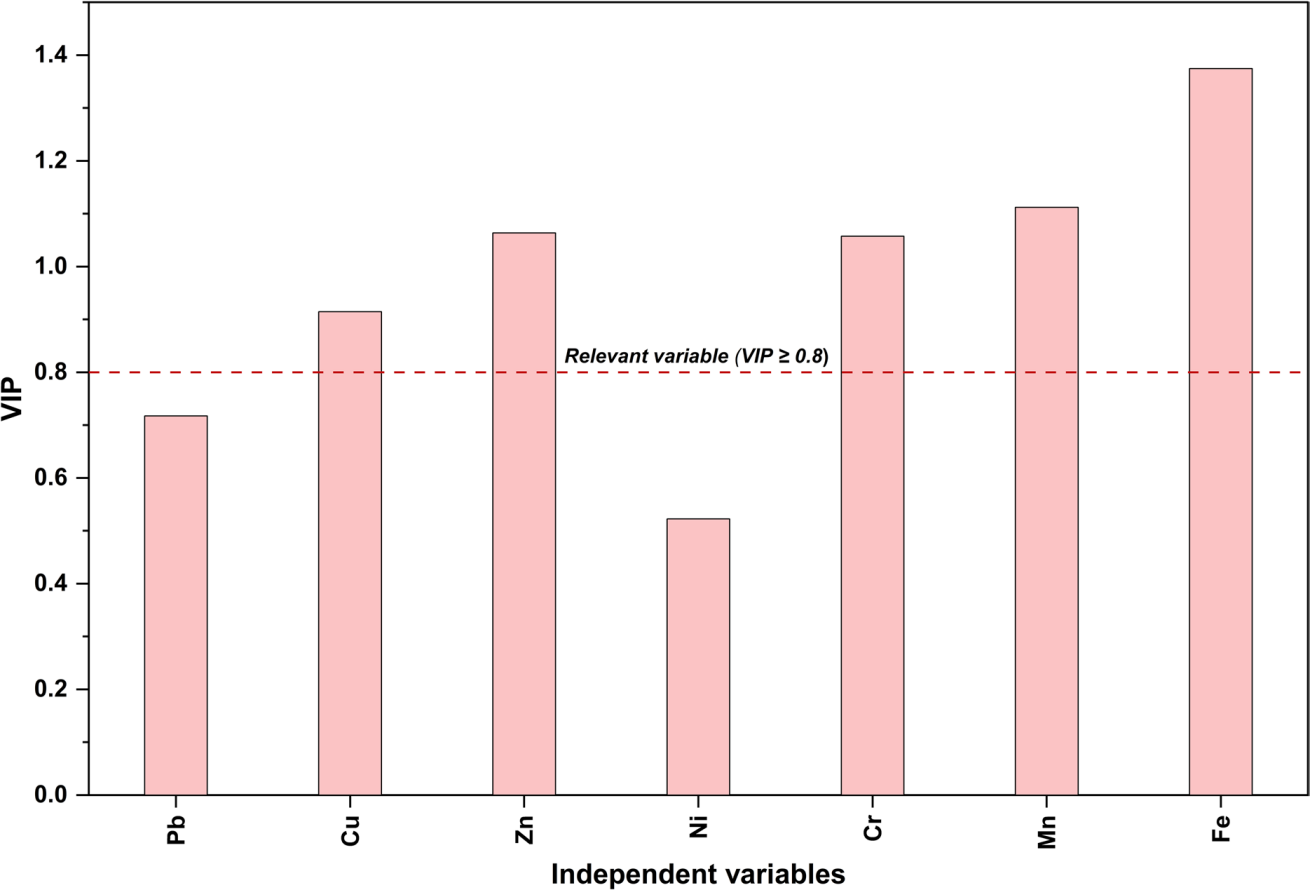
Variable Importance in Projection (VIP) scores derived from the PLS-DA model, illustrating the relative contribution of each PTE to zone differentiation

The Variable Importance in Projection (VIP) analysis identified five PTEs that exceeded the relevance threshold (VIP ≥ 0.8), including Fe (1.42), Cr (1.08), Zn (1.05), Mn (1.03), and Cu (0.90) (Fig. 15), establishing them as the primary geochemical indicators driving group separation as their influence, quantified by the standardized coefficients (Table S15), shaped the latent structure and supported categorization into distinct functional zones. Conversely, the low VIP scores of other PTEs, such as Ni (0.55), suggest insufficient spatial variability and operational specificity, rendering them non-discriminatory variables across the sample groups. This may also reflect their subordinate influence within the model, being overshadowed by more discriminatory and group-enriched PTEs, such as Fe and Mn in Category A samples and Cu in the cabin interiors (Group B1), which exerted a stronger influence on the classification structure.

#### Synthesis of multi-model geochemical discrimination

All models, with varying levels of precision, successfully identified certain groups as geochemically distinct, particularly Group B1 (drilling cabins) and Group B2 (external truck surfaces), which demonstrated high classification accuracy and minimal geochemical overlap. Meanwhile, Groups A1 (drilling zone), A2 (crushing sites), and A3 (unloading points) demonstrated some misclassifications across the MLR, DTC, and SVM models, which is meaningly considering that these groups belong to the same broader operational domain (Category A), where shared processes, spatial proximity, and dust dispersion mechanisms likely produce transitional or overlapping elemental signatures. Supporting this interpretation, PLS-DA revealed high leverage and residual patterns for samples from these zones, emphasizing intra-domain heterogeneity. Noteworthy, the variability observed between the training and testing accuracies of the DTC model reflects the similarity of mining-related processes operating under comparable environmental conditions across adjacent zones (A1, A2, and C), where shared emission sources and dust dispersion dynamics produce overlapping geochemical signatures.

Attaining maximal node-splitting importance in DTC, Cu and Pb were considered as dominant discriminators. This observation strongly supports their distinct anthropogenic origin, particularly from mechanical abrasion and fuel-derived residues associated with mining equipment and vehicular components. In contrast, Fe and Mn, typically associated with bulk ore handling and lithogenic sources, showed a more varied performance. Despite their negligible influence in the MLR model, as indicated by near-zero coefficients and ORs approximating unity, both elements exhibited strong relevance in PLS-DA, with VIP values exceeding the relevance threshold. This discrepancy underscores their latent role in geochemical differentiation; Fe and Mn, rather than delineating sharp inter-zone contrasts, may instead reflect more intricate compositional architectures embedded within the multivariate data space. This behavior implies that these elements contribute to shaping the underlying geochemical matrix, with their discriminative influence becoming evident only through dimensionality-reducing and structure-sensitive algorithms such as PLS-DA.

While unsupervised multivariate statistical methods (PCA and HCA) effectively delineated elemental associations and underlying potential geochemical processes (e.g., redox-driven mobilization), they showed limited ability to isolate functional-zone specificity. On the other hand, supervised machine learning models captured and operationalized these latent patterns into predictive classifications and actionable environmental insights, resolving nuanced inter-zone gradients and substantiating trace-element discriminators. For instance, the limitation of PCA to resolve transitional dust patterns between mechanistically related groups was explicitly rectified by supervised classifiers, which quantified misclassification probabilities and identified context-specific tracers (e.g., Cu and Pb). Moreover, supervised models enabled probabilistic interpretation, outlier detection, and performance ranking, capabilities critical for site-specific environmental evaluation. Recent comparable supervised classification frameworks have been successfully applied in similar geochemical contexts. For instance, Mangum et al. (2024) employed Linear Discriminant Analysis (LDA) to distinguish dust-emitting sites across arid regions of the western United States, demonstrating that geochemical signatures can differentiate sources more effectively than conventional unsupervised methods (e.g., PCA). Similarly, Sabbaghi (2024) applied SVM to recognize multi-element geochemical anomalies related to Pb–Zn mineralization in the Varcheh district, Iran, substantiating the reliability of the present modeling approach for environmental geochemical discrimination.

### Dust risk management and future perspectives

To foster environmental stewardship and inform evidence-based risk management in mitigating MD impacts within El-Gedida Mine, this study delineates six strategic pillars: operational control, engineering containment, dust suppression, occupational health safeguards, environmental monitoring, and eco-remediation. As a primary intervention, operational reconfigurations should prioritize the optimization of truck loading–unloading cycles, the reduction of vehicular idling durations, the phased renewal of the mine’s truck fleet with low-emission and sealed-cabin units, and the strict enforcement of low-speed driving protocols along haulage roads (Wang et al., 2023). Moreover, the integration of adaptive traffic scheduling frameworks synchronized with on-site meteorological sensors can enhance logistical coordination over vehicle movements and proactively minimize dust release during episodic wind events (Wang et al., 2024a, 2024b). Strategic implementation of engineering controls (e.g., enclosing milling circuits, transfer points, and crushers) alongside geochemically-informed zoning integrated into site layout planning effectively minimizes inter-zone contamination and strengthens source-specific mitigation (Lottermoser, 2017; Petavratzi et al., 2005). Building on these containment strategies, fine-scale dust suppression should target fugitive emissions at their operational origin. Water spray systems near crushing sites and vegetative windbreaks are recommended, as these measures have demonstrated attenuation of PM₁₀ and PM₂.₅ levels, achieving reductions exceeding 80% in controlled field studies (Duarte et al., 2022; Makkiabadi et al., 2022). To safeguard occupational health, it is imperative to enforce the mandatory use of PPE, implement targeted training programs, and ensure regular maintenance of machinery, particularly within confined microenvironments. Complementarily, the integration of smart PPE systems, featuring wearable particulate sensors and real-time compliance tracking (e.g., helmet- or vest-mounted PM monitors), is increasingly proposed, offering continuous exposure assessment and automated alerts that reinforce protocol adherence in high-risk zones (Formisano et al., 2024; Moon & Ju, 2024). Moreover, environmental monitoring should adopt passive dust gauges and emission factor analyses to evaluate long-term particulate deposition and pinpoint spatial emission hotspots. Enhancing these tools with IoT-based real-time sensors and machine-learning dispersion models enables high-resolution plume tracking and predictive critical emission points mapping (Tripathi et al., 2024). As a long-term stabilization measure, deploying eco-remediation, such as native hyperaccumulator species (e.g., *Conyza bonariensis*), offers a sustainable and cost-effective strategy for in-situ rehabilitation of metal-enriched environmental surfaces (e.g., nearby surface soil) affected by sustained particulate loading (Rizwan et al., 2019).

Building on the framework demonstrated in this study, extending this approach to other high-risk anthropogenic settings (e.g., cement industries, informal waste recycling hubs, and traffic-intensive urban areas) will broaden the environmental applicability of geochemical fingerprinting. Future investigations should also adopt denser, temporally stratified sampling strategies to capture subtle fluctuations in dust geochemistry driven by operational variability and seasonal climatic conditions. In parallel, the incorporation of explainable AI tools (e.g., SHAP or LIME) could enhance model interpretability and facilitate transparent communication of environmental risks to stakeholders (Alizamir et al., 2024; Talukdar et al., 2024). To translate these insights into actionable interventions, strategic collaboration of environmental geochemists with AI developers, occupational hygienists, and geospatial analysts is essential for transforming geochemical data into decision-support tools that guide targeted dust mitigation and contribute to sustainable mining.

## Conclusions

This study redefined mineral dust as a dynamic geochemical proxy that captures the operational characteristics and anthropogenic influences of distinct microenvironmental contexts within the mining environment. In this regard, an integrative approach was applied at El-Gedida Iron Mine, encompassing structured sampling, geochemical characterization, multivariate statistical analysis, and supervised machine learning to elucidate compositional variability, functional heterogeneity, and PTE-specific geochemical fingerprints across each mining setting. While Zn contamination appeared relatively widespread and uniformly distributed across all operational zones (CV = 10.37%), Cu exhibited a higher spatial variability (CV = 142.60%) with nearly a 40-fold concentration range, reflecting localized anthropogenic inputs. Supervised machine learning combined with multivariate statistics delineated two dominant geochemical fingerprints: a Cu–Pb signature associated with confined machinery cabins (Group B1), linked to mechanical abrasion and particle entrapment under restricted ventilation; and a Fe–Mn signature characterizing ore-handling zones (Category A), reflecting their direct association with the primary ore material and mobilization via intensive mechanical processing of Mn-rich iron ores. The MLR model achieved the highest prediction accuracy (95.8%), followed by the DTC model, which attained 83.3% during training. The PLS-DA model accounted for 71.6% of predictor variance and 65.3% of response variance, indicating reasonable discriminative performance. In contrast, the SVM yielded the lowest classification accuracy (41.67%), reflecting limited predictive capability. Composite pollution indices revealed the highest contaminant levels within the enclosed cabin environments (PLI = 3.45 and C_deg_ = 49.42), in contrast to the lower values observed on external truck surfaces (PLI = 2.25 and C_deg_ 28.40), where ambient open-pit conditions reduced particulate accumulation and surface adhesion. These findings offer actionable insights into implementing mitigation strategies tailored to the unique contamination signatures of each microenvironment. Despite the sample size and the geochemical overlap among spatially contiguous mining zones, the study achieved notably high classification accuracies and interpretable model behavior. Thus, future research should expand toward larger, temporally stratified datasets and integrate explainable AI frameworks to strengthen predictive reliability in complex mining geochemical systems. Additionally, future investigations should also prioritize the application of biomonitoring strategies employing biological matrices (e.g., blood, urine, and hair) to quantify internal burdens of PTEs, characterize their biokinetic behavior, and establish exposure–effect relationships among exposed mine workers. Furthermore, proactive operational models (e.g., load-handling cycles and onsite traffic orchestration) should be implemented to assimilate real-time atmospheric parameters and incorporate dynamic feedback mechanisms for particulate emissions, thereby enabling responsive, evidence-based decisions that advance environmentally optimized and worker-centered mining practices.

## Supplementary Information

Below is the link to the electronic supplementary material.

Supplementary file1 (DOCX 103 KB)

## Data Availability

All data generated or analyzed during this study are included in this published article and its supplementary information file.
