# Supplementary material for: Machine learning-driven geochemical fingerprinting and risk characterization of mineral dust across different operational settings in El-Gedida Iron Mine, Egypt
Source: Environ Geochem Health. 2025 Nov 17;47(12):575. doi: 10.1007/s10653-025-02850-w (PMC12628413; doi:10.1007/s10653-025-02850-w)
Supplement: Supplementary file 1 — Supplementary file1 (DOCX 103 KB) [file 10653_2025_2850_MOESM1_ESM.docx]

**Supplementary materials**

**Journal of Environmental Geochemistry and Health**

**Machine Learning-driven Geochemical Fingerprinting and Risk Characterization of Mineral Dust across Different Operational Settings in El-Gedida Iron Mine, Egypt**

| **Table S1** Categorization of mineral dust samples (F1–F24) collected across site-specific operational contexts and functional environmental surfaces within the El-Gedida iron mine site | | | | |
| --- | --- | --- | --- | --- |
| **Sample categories and groups** | | **Description** | **Sample codes** | **Site location** |
| **Category A: Operational zones** | | | | |
| **Group A1** | Surface drilling sites | Designated sites for conducting surface drilling operations. | F1 – F4 | 28°26'52"N, 29°11'24"E |
| **Group A2** | Crushing & grinding facilities | Facilities comprising crushing units and grinding mills for ore processing. | F5 – F8 | 28°28'01.9"N, 29°11'20.5" E |
| **Group A3** | Transport & unloading points | Designated locations for the loading and unloading of materials, such as loading docks. | F9 – F12 | 28°27'52" N, 29°11'03" E |
| **Category B: Equipment interior environments & exterior surfaces** | | | | |
| **Group B1** | Drilling cabins & heavy machinery interiors | Interiors of drilling cabins and heavy machinery, including loader cabins. | F13 – F16 | Non-stationary spatial positions |
| **Group B2** | Transport truck external surfaces | External surfaces of transport trucks exposed to particulate deposition. | F17 – F20 |  |
| **Category C: Dust accumulation sites on pathways** | | | | |
| **Group C** | Mine interior pathways | Pathways utilized for the transportation of workers and equipment, where dust accumulates. | F21 – F24 | 28°27'19"N, 29°11'23"E |

| **Table S2** Microwave-assisted digestion program for mineral dust samples | | | | | |
| --- | --- | --- | --- | --- | --- |
| Step | Target Temperature (°C) | Ramp Time  (min) | Hold Time (min) | Cooling (min) | Description |
| 1 | 200 | 15 | --- | --- | Controlled heating and acid activation |
| 2 | 200 | --- | 15 | --- | Matrix decomposition and element release |
| 3 | 85 | --- | --- | 15 | Cooling and solution stabilization |
| - The digestion was performed using a Milestone ETHOS EASY microwave system equipped with PTFE-TFM vessels rated for up to 100 bar and 300 °C. - The system automatically regulated pressure throughout the run, maintaining safe limits below 80 bar. | | | | | |

| **Table S3** Classification framework for the Geoaccumulation Index (I_geo_) and Contamination Factor (CF) (Hakanson, 1980; Iwegbue et al., 2024; Mostafa et al., 2024; Muller, 1969; Yesilkanat & Kobya, 2021) | | | | | |
| --- | --- | --- | --- | --- | --- |
| **Geoaccumulation Index (I_geo_)** | | | **Contamination Factor (CF)** | | |
| **Class** | **I_geo_ values** | **Contamination status** | **Class** | **CF values** | **Contamination level** |
| 0 | ≤ 0 | Uncontaminated | 1 | ≤ 1 | Low contamination |
| 1 | 0-1 | Uncontaminated to moderately contaminated | 2 | 1-3 | Moderate contamination |
| 2 | 1-2 | Moderately contaminated |  |  |  |
| 3 | 2-3 | Moderately contaminated to heavily contaminated | 3 | 3-6 | Considerable contamination |
| 4 | 3-4 | Heavily contaminated |  |  |  |
| 5 | 4-5 | Heavily contaminated to extremely contaminated | 4 | > 6 | Very high contamination |
| 6 | $>5$ | Extremely contaminated |  |  |  |

| **Table S4** Individual Ecological Risk Factor (Er) and Overall Ecological Risk Index (RI) classification categories (Guo et al., 2010; Hakanson, 1980; Ren et al., 2024; Yuan et al., 2025) | | | |
| --- | --- | --- | --- |
| **Er values** | **Classification** | **RI values** | **Classification** |
| < 40 | Low risk | < 150 | Low risk |
| 40-80 | Moderate risk | 150-300 | Moderate risk |
| 80-160 | Considerable risk | 300-600 | Considerable risk |
| 160-320 | High risk | ≥ 600 | High risk |
| ≥ 320 | Very high risk |  |  |

| **Table S5** Description of exposure parameters used for ADD calculations | | | | |
| --- | --- | --- | --- | --- |
| **Symbol (unit)** | **Parameter** | **Value** | | **Reference** |
|  |  | **Children** | **Adults** |  |
| IR_Ing_ (mg/d) | Ingestion rate | 200 | 100 | (USEPA, 1996, 2002) |
| ED (yr) | Exposure duration | 6 | 24 | (USEPA, 2002) |
| EF (d/yr) | Exposure frequency | 350 | | (USEPA, 1996) |
| BW (kg) | Average body weight | 15 | 70 | (USEPA, 2002) |
| AT (d) | Averaging time | 365 × ED | | (Dat et al., 2021; Malakootian et al., 2021) |
| IR_Inh_ (m^3^/d) | Inhalation rate | 7.63 | 20 | (Ferreira-Baptista & De Miguel, 2005; USEPA, 2002; Xu et al., 2013) |
| PEF (m^3^/kg) | Particle emission factor | 1.36×10^9^ | | (USEPA, 1996, 2002, 2011) |
| SA (cm^2^) | Exposed skin surface area | 2800 | 5700 | (Dat et al., 2021; USEPA, 2002) |
| AF (mg/cm^2^/d) | Skin-soil adherence factor | 0.2 | 0.07 | (USEPA, 2002) |
| DAF (unit-less) | Dermal absorption factor | 0.001 | | (USEPA, 2002) |
| CF (kg/mg) | Conversion factor | 1$\times$10^-6^ | | (USEPA, 2002) |

| **Table S6** Reference Doses (RfDs) and Cancer Slope Factors (CSFs) for PTEs via different exposure pathways (Adimalla, 2020; Al-Shidi et al., 2021; Cunha-Lopes et al., 2022; Dat et al., 2021; Malakootian et al., 2021; Tan et al., 2018; USEPA, 2002, 2005, 2011, 2012) | | | | |
| --- | --- | --- | --- | --- |
| **PTEs** | **RfD (mg/kg/day)** | | | **CSF (mg/kg/day)** |
|  | Ingestion | Inhalation | Dermal contact |  |
| Pb | 3.50E-03 | 3.52E-03 | 5.25E-04 | 8.50E-03 |
| Cu | 4.00E-02 | 4.02E-02 | 1.20E-02 | - |
| Zn | 3.00E-01 | 3.00E-01 | 6.00E-02 | - |
| Ni | 2.00E-02 | 2.06E-02 | 5.40E-03 | 8.40E-01 |
| Cr | 3.00E-03 | 2.86E-05 | 6.00E-05 | 5.00E-01 |

| **Table S7** Shapiro–Wilk normality test results for PTE concentrations in mineral dust samples at El-Gedida iron mine | | | | |
| --- | --- | --- | --- | --- |
| **PTEs** | **Sample group** | | **W (Shapiro–Wilk)** | ***p*-value** |
| Pb | A1 | Surface drilling sites | 0.944709 | 0.683235 |
|  | A2 | Crushing & grinding facilities | 0.931601 | 0.603843 |
|  | A3 | Transport & unloading points | 0.97061 | 0.84526 |
|  | B1 | Drilling cabins & heavy machinery interiors | 0.955018 | 0.747566 |
|  | B2 | Transport truck external surfaces | 0.977001 | 0.884223 |
|  | C | Mine interior pathways | 0.997598 | 0.992153 |
| Cu | A1 | Surface drilling sites | 0.963612 | 0.801618 |
|  | A2 | Crushing & grinding facilities | 0.940794 | 0.659175 |
|  | A3 | Transport & unloading points | 0.894242 | 0.403024 |
|  | B1 | Drilling cabins & heavy machinery interiors | 0.840276 | 0.196209 |
|  | B2 | Transport truck external surfaces | 0.914358 | 0.505795 |
|  | C | Mine interior pathways | 0.981437 | 0.910395 |
| Zn | A1 | Surface drilling sites | 0.864066 | 0.275034 |
|  | A2 | Crushing & grinding facilities | 0.854743 | 0.241895 |
|  | A3 | Transport & unloading points | 0.94411 | 0.679539 |
|  | B1 | Drilling cabins & heavy machinery interiors | 0.943904 | 0.678271 |
|  | B2 | Transport truck external surfaces | 0.942602 | 0.670252 |
|  | C | Mine interior pathways | 0.876637 | 0.324473 |
| Ni | A1 | Surface drilling sites | 0.946491 | 0.69427 |
|  | A2 | Crushing & grinding facilities | 0.816265 | 0.134733 |
|  | A3 | Transport & unloading points | 0.936358 | 0.632249 |
|  | B1 | Drilling cabins & heavy machinery interiors | 0.994324 | 0.978435 |
|  | B2 | Transport truck external surfaces | 0.98968 | 0.955822 |
|  | C | Mine interior pathways | 0.9823 | 0.915375 |
| Cr | A1 | Surface drilling sites | 0.767884 | 0.055993 |
|  | A2 | Crushing & grinding facilities | 0.821992 | 0.147867 |
|  | A3 | Transport & unloading points | 0.864733 | 0.277517 |
|  | B1 | Drilling cabins & heavy machinery interiors | 0.995787 | 0.984861 |
|  | B2 | Transport truck external surfaces | 0.977174 | 0.885258 |
|  | C | Mine interior pathways | 0.903581 | 0.449062 |
| Mn | A1 | Surface drilling sites | 0.836537 | 0.185496 |
|  | A2 | Crushing & grinding facilities | 0.97614 | 0.879047 |
|  | A3 | Transport & unloading points | 0.94528 | 0.686769 |
|  | B1 | Drilling cabins & heavy machinery interiors | 0.94327 | 0.674363 |
|  | B2 | Transport truck external surfaces | 0.868645 | 0.292404 |
|  | C | Mine interior pathways | 0.968253 | 0.830634 |
| Fe | A1 | Surface drilling sites | 0.822109 | 0.148144 |
|  | A2 | Crushing & grinding facilities | 0.95624 | 0.755249 |
|  | A3 | Transport & unloading points | 0.887627 | 0.372236 |
|  | B1 | Drilling cabins & heavy machinery interiors | 0.848104 | 0.220078 |
|  | B2 | Transport truck external surfaces | 0.952246 | 0.730168 |
|  | C | Mine interior pathways | 0.872821 | 0.308882 |
| values of *p* > 0.05 indicate no significant deviation from normality | | | | |

| **Table S8** Contamination Factors (CFs) of PTEs in mineral dust from El-Gedida iron mine, Western Desert, Egypt | | | | | | | |
| --- | --- | --- | --- | --- | --- | --- | --- |
| **Samples** | **CF** | | | | | | |
|  | **Pb** | **Cu** | **Zn** | **Ni** | **Cr** | **Mn** | **Fe** |
| A) Operational zones | | | | | | | |
| A1) Surface drilling sites | | | | | | | |
| F1 | 3.68 | 0.20 | 3.01 | 0.27 | 1.09 | 24.64 | 5.83 |
| F2 | 3.90 | 0.27 | 3.42 | 0.30 | 1.31 | 32.24 | 6.70 |
| F3 | 3.34 | 0.24 | 3.00 | 0.32 | 1.10 | 26.62 | 5.76 |
| F4 | 3.43 | 0.21 | 2.84 | 0.25 | 1.05 | 25.67 | 5.57 |
| Mean | 3.59 | 0.23 | 3.07 | 0.29 | 1.14 | 27.29 | 5.97 |
| A2) Crushing & grinding facilities | | | | | | | |
| F5 | 3.98 | 0.20 | 3.10 | 0.27 | 1.12 | 27.72 | 6.27 |
| F6 | 4.25 | 0.24 | 3.73 | 0.35 | 1.38 | 31.40 | 7.45 |
| F7 | 4.06 | 0.18 | 3.07 | 0.29 | 1.15 | 26.60 | 6.35 |
| F8 | 3.55 | 0.21 | 2.86 | 0.27 | 1.07 | 21.61 | 5.40 |
| Mean | 3.96 | 0.21 | 3.19 | 0.30 | 1.18 | 26.83 | 6.37 |
| A3) Transport & unloading points | | | | | | | |
| F9 | 1.01 | 0.22 | 3.00 | 0.26 | 0.84 | 37.61 | 4.54 |
| F10 | 1.32 | 0.13 | 3.31 | 0.26 | 0.84 | 35.47 | 5.62 |
| F11 | 1.14 | 0.16 | 3.01 | 0.23 | 0.75 | 33.54 | 4.88 |
| F12 | 1.16 | 0.14 | 2.74 | 0.24 | 0.72 | 28.14 | 4.83 |
| Mean | 1.16 | 0.16 | 3.02 | 0.25 | 0.79 | 33.69 | 4.97 |
| B) Equipment interior environments & exterior surfaces | | | | | | | |
| B1) Drilling cabins & heavy machinery interiors | | | | | | | |
| F13 | 3.91 | 3.80 | 3.12 | 0.42 | 1.44 | 28.88 | 5.01 |
| F14 | 4.63 | 3.33 | 3.38 | 0.48 | 1.59 | 27.50 | 5.35 |
| F15 | 5.06 | 3.76 | 3.82 | 0.54 | 1.80 | 31.07 | 6.04 |
| F16 | 2.79 | 5.21 | 2.97 | 0.35 | 1.27 | 35.19 | 4.98 |
| Mean | 4.10 | 4.03 | 3.32 | 0.45 | 1.53 | 30.66 | 5.35 |
| B2) Transport truck external surfaces | | | | | | | |
| F17 | 2.68 | 1.19 | 2.73 | 0.32 | 1.42 | 15.72 | 4.59 |
| F18 | 2.57 | 0.78 | 3.00 | 0.33 | 1.50 | 17.22 | 4.71 |
| F19 | 2.84 | 1.63 | 2.49 | 0.30 | 1.32 | 14.25 | 4.48 |
| F20 | 2.95 | 1.55 | 2.57 | 0.35 | 1.37 | 14.22 | 4.52 |
| Mean | 2.76 | 1.29 | 2.70 | 0.33 | 1.40 | 15.35 | 4.58 |
| C) Dust accumulation sites on pathways (mine interior pathways) | | | | | | | |
| F21 | 3.21 | 0.28 | 3.07 | 0.27 | 1.15 | 30.09 | 6.11 |
| F22 | 3.46 | 0.24 | 2.88 | 0.26 | 1.13 | 27.50 | 5.68 |
| F23 | 3.04 | 0.25 | 2.87 | 0.25 | 1.06 | 29.17 | 5.68 |
| F24 | 3.31 | 0.23 | 2.96 | 0.29 | 1.12 | 26.17 | 5.89 |
| Mean | 3.26 | 0.25 | 2.95 | 0.27 | 1.12 | 28.23 | 5.84 |
| Statistical summary | | | | | | | |
| Maximum | 5.06 | 5.21 | 3.82 | 0.54 | 1.80 | 37.61 | 7.45 |
| Minimum | 1.01 | 0.13 | 2.49 | 0.23 | 0.72 | 14.22 | 4.48 |
| Mean | 3.14 | 1.03 | 3.04 | 0.31 | 1.19 | 27.01 | 5.51 |
| Standard deviation | 1.09 | 1.46 | 0.32 | 0.08 | 0.26 | 6.47 | 0.76 |

| **Table S9** Geoaccumulation Index (I_geo_) values for PTEs in mineral dust from El-Gedida iron mine, Western Desert, Egypt | | | | | | | |
| --- | --- | --- | --- | --- | --- | --- | --- |
| **Samples** | **I_geo_** | | | | | | |
|  | **Pb** | **Cu** | **Zn** | **Ni** | **Cr** | **Mn** | **Fe** |
| A) Operational zones | | | | | | | |
| A1) Surface drilling sites | | | | | | | |
| F1 | 1.29 | -2.91 | 1.00 | -2.46 | -0.46 | 4.04 | 1.96 |
| F2 | 1.38 | -2.49 | 1.19 | -2.31 | -0.19 | 4.43 | 2.16 |
| F3 | 1.16 | -2.66 | 1.00 | -2.25 | -0.45 | 4.15 | 1.94 |
| F4 | 1.19 | -2.82 | 0.92 | -2.56 | -0.51 | 4.10 | 1.89 |
| Mean | 1.26 | -2.72 | 1.03 | -2.40 | -0.40 | 4.18 | 1.99 |
| A2) Crushing & grinding facilities | | | | | | | |
| F5 | 1.41 | -2.92 | 1.05 | -2.45 | -0.42 | 4.21 | 2.06 |
| F6 | 1.50 | -2.63 | 1.32 | -2.12 | -0.12 | 4.39 | 2.31 |
| F7 | 1.44 | -3.04 | 1.03 | -2.36 | -0.39 | 4.15 | 2.08 |
| F8 | 1.24 | -2.86 | 0.93 | -2.47 | -0.48 | 3.85 | 1.85 |
| Mean | 1.40 | -2.86 | 1.08 | -2.35 | -0.35 | 4.15 | 2.08 |
| A3) Transport & unloading points | | | | | | | |
| F9 | -0.57 | -2.79 | 1.00 | -2.56 | -0.84 | 4.65 | 1.60 |
| F10 | -0.18 | -3.53 | 1.14 | -2.52 | -0.83 | 4.56 | 1.90 |
| F11 | -0.40 | -3.22 | 1.00 | -2.70 | -1.00 | 4.48 | 1.70 |
| F12 | -0.37 | -3.39 | 0.87 | -2.65 | -1.07 | 4.23 | 1.69 |
| Mean | -0.38 | -3.23 | 1.00 | -2.61 | -0.94 | 4.48 | 1.72 |
| B) Equipment interior environments & exterior surfaces | | | | | | | |
| B1) Drilling cabins & heavy machinery interiors | | | | | | | |
| F13 | 1.38 | 1.34 | 1.06 | -1.83 | -0.06 | 4.27 | 1.74 |
| F14 | 1.63 | 1.15 | 1.17 | -1.64 | 0.08 | 4.20 | 1.83 |
| F15 | 1.75 | 1.33 | 1.35 | -1.46 | 0.26 | 4.37 | 2.01 |
| F16 | 0.89 | 1.80 | 0.99 | -2.09 | -0.24 | 4.55 | 1.73 |
| Mean | 1.41 | 1.41 | 1.14 | -1.76 | 0.01 | 4.35 | 1.83 |
| B2) Transport truck external surfaces | | | | | | | |
| F17 | 0.84 | -0.33 | 0.86 | -2.24 | -0.08 | 3.39 | 1.61 |
| F18 | 0.78 | -0.94 | 1.00 | -2.17 | 0.00 | 3.52 | 1.65 |
| F19 | 0.92 | 0.12 | 0.73 | -2.33 | -0.18 | 3.25 | 1.58 |
| F20 | 0.97 | 0.04 | 0.78 | -2.11 | -0.13 | 3.25 | 1.59 |
| Mean | 0.88 | -0.28 | 0.84 | -2.21 | -0.10 | 3.35 | 1.61 |
| C) Dust accumulation sites on pathways (mine interior pathways) | | | | | | | |
| F21 | 1.10 | -2.43 | 1.03 | -2.49 | -0.39 | 4.33 | 2.03 |
| F22 | 1.20 | -2.63 | 0.94 | -2.51 | -0.41 | 4.20 | 1.92 |
| F23 | 1.02 | -2.56 | 0.94 | -2.61 | -0.51 | 4.28 | 1.92 |
| F24 | 1.14 | -2.72 | 0.98 | -2.39 | -0.43 | 4.13 | 1.97 |
| Mean | 1.12 | -2.59 | 0.97 | -2.50 | -0.44 | 4.24 | 1.96 |
| Statistical summary | | | | | | | |
| Maximum | 1.75 | 1.80 | 1.35 | -1.46 | 0.26 | 4.65 | 2.31 |
| Minimum | -0.57 | -3.53 | 0.73 | -2.70 | -1.07 | 3.25 | 1.58 |
| Mean | 0.95 | -1.71 | 1.01 | -2.30 | -0.37 | 4.12 | 1.86 |
| Standard deviation | 0.65 | 1.75 | 0.15 | 0.31 | 0.33 | 0.40 | 0.20 |

| **Table S10** Composite pollution assessment of PTEs in mineral dust from El-Gedida iron mine, Western Desert, Egypt | | | |
| --- | --- | --- | --- |
| **Samples** | **Composite pollution indices** | | |
|  | **PLI** | **C_deg_** | **NPI** |
| A) Operational zones | | | |
| A1) Surface drilling sites | | | |
| F1 | 1.91 | 38.72 | 9.54 |
| F2 | 2.26 | 48.14 | 12.46 |
| F3 | 2.00 | 40.38 | 10.29 |
| F4 | 1.86 | 39.02 | 9.93 |
| Mean | 2.01 | 41.57 | 10.56 |
| A2) Crushing & grinding facilities | | | |
| F5 | 2.00 | 42.66 | 10.73 |
| F6 | 2.38 | 48.80 | 12.16 |
| F7 | 2.00 | 41.70 | 10.30 |
| F8 | 1.84 | 34.97 | 8.38 |
| Mean | 2.06 | 42.03 | 10.39 |
| A3) Transport & unloading points | | | |
| F9 | 1.58 | 47.48 | 14.44 |
| F10 | 1.58 | 46.95 | 13.64 |
| F11 | 1.48 | 43.71 | 12.89 |
| F12 | 1.40 | 37.97 | 10.83 |
| Mean | 1.51 | 44.03 | 12.95 |
| B) Equipment interior environments & exterior surfaces | | | |
| B1) Drilling cabins & heavy machinery interiors | | | |
| F13 | 3.28 | 46.58 | 11.20 |
| F14 | 3.45 | 46.26 | 10.69 |
| F15 | 3.88 | 52.09 | 12.08 |
| F16 | 3.19 | 52.76 | 13.60 |
| Mean | 3.45 | 49.42 | 11.89 |
| B2) Transport truck external surfaces | | | |
| F17 | 2.24 | 28.65 | 6.14 |
| F18 | 2.19 | 30.11 | 6.71 |
| F19 | 2.25 | 27.31 | 5.58 |
| F20 | 2.32 | 27.53 | 5.58 |
| Mean | 2.25 | 28.40 | 6.00 |
| C) Dust accumulation sites on pathways (mine interior pathways) | | | |
| F21 | 2.06 | 44.18 | 11.62 |
| F22 | 1.96 | 41.15 | 10.63 |
| F23 | 1.92 | 42.32 | 11.26 |
| F24 | 1.96 | 39.97 | 10.12 |
| Mean | 1.98 | 41.91 | 10.91 |
| Statistical summary | | | |
| Maximum | 3.88 | 52.76 | 14.44 |
| Minimum | 1.40 | 27.31 | 5.58 |
| Mean | 2.21 | 41.23 | 10.45 |
| Standard deviation | 0.63 | 7.30 | 2.47 |

| **Table S11** Posterior class membership probabilities for dust samples as estimated by the Multinomial Logistic Regression (MLR) model | | | | | | | |
| --- | --- | --- | --- | --- | --- | --- | --- |
| **Samples** | **Estimated Response Probabilities** | | | | | | **Predicted Membership** |
|  | **A1** | **A2** | **A3** | **B1** | **B2** | **C** |  |
| F1 | 4.41E-01 | 5.48E-01 | 2.35E-03 | 4.80E-03 | 2.77E-03 | 2.10E-04 | A2 |
| F2 | 9.25E-01 | 3.14E-02 | 7.03E-03 | 3.66E-03 | 2.02E-03 | 3.07E-02 | A1 |
| F3 | 9.02E-01 | 1.19E-02 | 2.12E-02 | 9.00E-03 | 5.10E-03 | 5.12E-02 | A1 |
| F4 | 7.89E-01 | 9.19E-02 | 1.18E-02 | 9.76E-03 | 9.94E-03 | 8.80E-02 | A1 |
| F5 | 2.71E-01 | 7.17E-01 | 1.08E-03 | 3.48E-03 | 9.55E-04 | 5.81E-03 | A2 |
| F6 | 9.01E-02 | 9.07E-01 | 6.65E-04 | 1.58E-03 | 9.73E-04 | 1.22E-04 | A2 |
| F7 | 2.91E-02 | 9.36E-01 | 1.66E-04 | 1.10E-03 | 7.81E-04 | 3.30E-02 | A2 |
| F8 | 4.15E-01 | 5.75E-01 | 1.66E-03 | 3.95E-03 | 4.27E-03 | 3.23E-05 | A2 |
| F9 | 2.38E-01 | 1.00E-16 | 7.62E-01 | 2.37E-06 | 7.68E-08 | 3.07E-07 | A3 |
| F10 | 4.13E-02 | 2.30E-12 | 9.59E-01 | 3.25E-05 | 9.01E-06 | 2.08E-07 | A3 |
| F11 | 8.06E-02 | 3.92E-13 | 9.19E-01 | 2.50E-05 | 4.09E-06 | 1.44E-07 | A3 |
| F12 | 1.17E-02 | 4.67E-10 | 9.85E-01 | 2.79E-04 | 1.32E-03 | 1.71E-03 | A3 |
| F13 | 7.02E-04 | 1.64E-05 | 7.56E-04 | 9.98E-01 | 7.37E-04 | 1.33E-06 | B1 |
| F14 | 6.60E-02 | 2.69E-03 | 3.19E-04 | 9.31E-01 | 1.46E-04 | 2.65E-09 | B1 |
| F15 | 3.92E-02 | 1.55E-03 | 1.23E-04 | 9.59E-01 | 2.90E-05 | 2.76E-11 | B1 |
| F16 | 2.13E-08 | 1.48E-10 | 1.45E-03 | 9.93E-01 | 2.28E-03 | 3.27E-03 | B1 |
| F17 | 7.74E-05 | 6.11E-04 | 1.54E-04 | 4.12E-04 | 9.98E-01 | 2.93E-04 | B2 |
| F18 | 1.19E-02 | 2.78E-04 | 3.08E-03 | 6.17E-04 | 9.84E-01 | 1.46E-06 | B2 |
| F19 | 1.08E-06 | 2.64E-03 | 1.15E-05 | 4.72E-04 | 9.84E-01 | 1.33E-02 | B2 |
| F20 | 1.11E-05 | 5.03E-03 | 3.46E-05 | 1.22E-03 | 9.83E-01 | 1.05E-02 | B2 |
| F21 | 1.52E-02 | 2.84E-04 | 3.07E-03 | 5.40E-04 | 2.17E-03 | 9.79E-01 | C |
| F22 | 1.73E-01 | 5.62E-03 | 3.25E-03 | 1.79E-03 | 2.91E-03 | 8.13E-01 | C |
| F23 | 1.78E-02 | 6.08E-05 | 3.21E-03 | 3.90E-04 | 1.02E-03 | 9.78E-01 | C |
| F24 | 9.70E-02 | 3.23E-02 | 8.32E-03 | 5.01E-03 | 2.20E-02 | 8.35E-01 | C |

| **Table S12** Multinomial Logistic Regression (MLR) coefficients, standard errors, and odds ratios for geochemical predictors across site-specific operational contexts and functional environmental surfaces | | | | | | | | | | | | | |
| --- | --- | --- | --- | --- | --- | --- | --- | --- | --- | --- | --- | --- | --- |
| Groups | PTEs | Coefficients (β) | Standard Error | Wald Test | Degrees of Freedom | t-Value | Prob>\|t\| | Conclusion  at Level 5% | 95% LCL for Coefficients | 95% UCL for Coefficients | Odds Ratio | 95% LCL for Odds Ratio | 95% UCL for Odds Ratio |
| **A1** | *Intercept* | 17.5853 | 25.8536 | 0.4627 | 1 | -33.09 | 68.26 | NS | -33.0868 | 68.25737 | -- | -- | -- |
|  | Pb | -0.4988 | 0.6213 | 0.6446 | 1 | -1.72 | 0.72 | NS | -1.71657 | 0.718899 | 6.07E-01 | 1.80E-01 | 2.05E+00 |
|  | Cu | 0.1187 | 0.3709 | 0.1024 | 1 | -0.61 | 0.85 | NS | -0.60818 | 0.845535 | 1.13E+00 | 5.44E-01 | 2.33E+00 |
|  | Zn | 0.3543 | 0.3308 | 1.1470 | 1 | -0.29 | 1.00 | NS | -0.29412 | 1.002749 | 1.43E+00 | 7.45E-01 | 2.73E+00 |
|  | Ni | -0.4270 | 2.4624 | 0.0301 | 1 | -5.25 | 4.40 | NS | -5.25318 | 4.399112 | 6.52E-01 | 5.23E-03 | 8.14E+01 |
|  | Cr | -0.4348 | 0.5829 | 0.5564 | 1 | -1.58 | 0.71 | NS | -1.57719 | 0.707648 | 6.47E-01 | 2.07E-01 | 2.03E+00 |
|  | Mn | 0.0014 | 0.0016 | 0.7517 | 1 | 0.00 | 0.00 | NS | -0.0018 | 0.004657 | 1.00E+00 | 9.98E-01 | 1.00E+00 |
|  | Fe | -0.0002 | 0.0004 | 0.3521 | 1 | 0.00 | 0.00 | NS | -0.00097 | 0.000518 | 1.00E+00 | 9.99E-01 | 1.00E+00 |
| **A3** | *Intercept* | 22.6345 | 47.2918 | 0.2291 | 1 | -70.06 | 115.32 | NS | -70.0556 | 115.3246 | 6.76E+09 | 3.76E-31 | 1.22E+50 |
|  | Pb | -0.8375 | 0.8090 | 1.0716 | 1 | -2.42 | 0.75 | NS | -2.42314 | 0.748159 | 4.33E-01 | 8.86E-02 | 2.11E+00 |
|  | Cu | 0.2123 | 0.4000 | 0.2816 | 1 | -0.57 | 1.00 | NS | -0.57174 | 0.996322 | 1.24E+00 | 5.65E-01 | 2.71E+00 |
|  | Zn | 0.2793 | 0.5624 | 0.2466 | 1 | -0.82 | 1.38 | NS | -0.82304 | 1.38162 | 1.32E+00 | 4.39E-01 | 3.98E+00 |
|  | Ni | -0.5714 | 3.2217 | 0.0315 | 1 | -6.89 | 5.74 | NS | -6.88588 | 5.742995 | 5.65E-01 | 1.02E-03 | 3.12E+02 |
|  | Cr | -0.3856 | 0.7527 | 0.2624 | 1 | -1.86 | 1.09 | NS | -1.86096 | 1.089718 | 6.80E-01 | 1.56E-01 | 2.97E+00 |
|  | Mn | 0.0013 | 0.0020 | 0.4414 | 1 | 0.00 | 0.01 | NS | -0.00263 | 0.005318 | 1.00E+00 | 9.97E-01 | 1.01E+00 |
|  | Fe | -0.0001 | 0.0005 | 0.0429 | 1 | 0.00 | 0.00 | NS | -0.00107 | 0.000868 | 1.00E+00 | 9.99E-01 | 1.00E+00 |
| **B1** | *Intercept* | 21.9211 | 43.5869 | 0.2529 | 1 | -63.51 | 107.35 | NS | -63.5075 | 107.3498 | 3.31E+09 | 2.62E-28 | 4.18E+46 |
|  | Pb | -0.7060 | 0.8668 | 0.6634 | 1 | -2.40 | 0.99 | NS | -2.40475 | 0.992852 | 4.94E-01 | 9.03E-02 | 2.70E+00 |
|  | Cu | 0.2908 | 0.4693 | 0.3841 | 1 | -0.63 | 1.21 | NS | -0.629 | 1.210696 | 1.34E+00 | 5.33E-01 | 3.36E+00 |
|  | Zn | 0.1661 | 0.8787 | 0.0357 | 1 | -1.56 | 1.89 | NS | -1.55616 | 1.888415 | 1.18E+00 | 2.11E-01 | 6.61E+00 |
|  | Ni | -0.3736 | 4.1377 | 0.0082 | 1 | -8.48 | 7.74 | NS | -8.4832 | 7.736103 | 6.88E-01 | 2.07E-04 | 2.29E+03 |
|  | Cr | -0.3909 | 1.0562 | 0.1370 | 1 | -2.46 | 1.68 | NS | -2.46089 | 1.679178 | 6.76E-01 | 8.54E-02 | 5.36E+00 |
|  | Mn | 0.0008 | 0.0036 | 0.0449 | 1 | -0.01 | 0.01 | NS | -0.00633 | 0.007867 | 1.00E+00 | 9.94E-01 | 1.01E+00 |
|  | Fe | 0.0000 | 0.0007 | 0.0005 | 1 | 0.00 | 0.00 | NS | -0.00139 | 0.001419 | 1.00E+00 | 9.99E-01 | 1.00E+00 |
| **B2** | *Intercept* | 32.4827 | 69.7372 | 0.2170 | 1 | -104.20 | 169.17 | NS | -104.2 | 169.1651 | 1.28E+14 | 5.58E-46 | 2.93E+73 |
|  | Pb | -0.9673 | 0.8627 | 1.2573 | 1 | -2.66 | 0.72 | NS | -2.65805 | 0.723498 | 3.80E-01 | 7.01E-02 | 2.06E+00 |
|  | Cu | 0.2649 | 0.4325 | 0.3749 | 1 | -0.58 | 1.11 | NS | -0.58292 | 1.112636 | 1.30E+00 | 5.58E-01 | 3.04E+00 |
|  | Zn | 0.0656 | 0.6779 | 0.0094 | 1 | -1.26 | 1.39 | NS | -1.2631 | 1.394299 | 1.07E+00 | 2.83E-01 | 4.03E+00 |
|  | Ni | -0.4322 | 3.7615 | 0.0132 | 1 | -7.80 | 6.94 | NS | -7.80452 | 6.940158 | 6.49E-01 | 4.08E-04 | 1.03E+03 |
|  | Cr | -0.2004 | 0.9307 | 0.0464 | 1 | -2.02 | 1.62 | NS | -2.02457 | 1.623672 | 8.18E-01 | 1.32E-01 | 5.07E+00 |
|  | Mn | 0.0003 | 0.0036 | 0.0072 | 1 | -0.01 | 0.01 | NS | -0.00683 | 0.007446 | 1.00E+00 | 9.93E-01 | 1.01E+00 |
|  | Fe | 0.0001 | 0.0006 | 0.0457 | 1 | 0.00 | 0.00 | NS | -0.00098 | 0.001222 | 1.00E+00 | 9.99E-01 | 1.00E+00 |
| **C** | *Intercept* | 50.9636 | 37.9079 | 1.8074 | 1 | -23.33 | 125.26 | NS | -23.3346 | 125.2618 | 1.36E+22 | 7.34E-11 | 2.51E+54 |
|  | Pb | -1.0609 | 0.6289 | 2.8456 | 1 | -2.29 | 0.17 | NS | -2.29359 | 0.171751 | 3.46E-01 | 1.01E-01 | 1.19E+00 |
|  | Cu | 0.2029 | 0.4110 | 0.2437 | 1 | -0.60 | 1.01 | NS | -0.60261 | 1.00835 | 1.22E+00 | 5.47E-01 | 2.74E+00 |
|  | Zn | -0.6751 | 0.5859 | 1.3280 | 1 | -1.82 | 0.47 | NS | -1.82341 | 0.473134 | 5.09E-01 | 1.61E-01 | 1.61E+00 |
|  | Ni | 0.6522 | 2.8652 | 0.0518 | 1 | -4.96 | 6.27 | NS | -4.96356 | 6.267896 | 1.92E+00 | 6.99E-03 | 5.27E+02 |
|  | Cr | 0.0914 | 0.6741 | 0.0184 | 1 | -1.23 | 1.41 | NS | -1.22983 | 1.412544 | 1.10E+00 | 2.92E-01 | 4.11E+00 |
|  | Mn | 0.0025 | 0.0021 | 1.4399 | 1 | 0.00 | 0.01 | NS | -0.00158 | 0.006582 | 1.00E+00 | 9.98E-01 | 1.01E+00 |
|  | Fe | 0.0005 | 0.0005 | 0.9821 | 1 | 0.00 | 0.00 | NS | -0.00048 | 0.001452 | 1.00E+00 | 1.00E+00 | 1.00E+00 |
| NS: Not statistically significant (*p* > 0.05) | | | | | | | | | | | | | |

| **Table S13** Confusion matrices for the Decision Tree Classifier (DTC) applied to the training and testing datasets | | | | | | | | | | | | | | | | |
| --- | --- | --- | --- | --- | --- | --- | --- | --- | --- | --- | --- | --- | --- | --- | --- | --- |
| **Zone** | **Predicted Class (Training)** | | | | | | | | **Predicted Class (Test)** | | | | | | | |
|  | Sample count | A1 | A2 | A3 | B1 | B2 | C | Accuracy (%) | Sample count | A1 | A2 | A3 | B1 | B2 | C | Accuracy (%) |
| A1 | 4 | 0 | 2 | 0 | 0 | 0 | 2 | 0 | 4 | 1 | 1 | 0 | 0 | 0 | 2 | 25 |
| A2 | 4 | 0 | 4 | 0 | 0 | 0 | 0 | 100 | 4 | 0 | 2 | 0 | 0 | 0 | 2 | 50 |
| A3 | 4 | 0 | 0 | 4 | 0 | 0 | 0 | 100 | 4 | 2 | 0 | 2 | 0 | 0 | 0 | 50 |
| B1 | 4 | 0 | 0 | 0 | 4 | 0 | 0 | 100 | 4 | 2 | 0 | 0 | 2 | 0 | 0 | 50 |
| B2 | 4 | 0 | 0 | 0 | 0 | 4 | 0 | 100 | 4 | 0 | 0 | 0 | 0 | 3 | 1 | 75 |
| C | 4 | 0 | 0 | 0 | 0 | 0 | 4 | 100 | 4 | 1 | 0 | 0 | 0 | 1 | 2 | 50 |
| Overall | 24 | 0 | 6 | 4 | 4 | 4 | 6 | 83.33 | 24 | 6 | 3 | 2 | 2 | 4 | 7 | 50 |

| **Table S14** Diagnostic statistics for all samples based on the fitted Partial Least Squares Discriminant Analysis (PLS-DA) model, including predicted continuous responses (fitted zone codes), distances in the X-space (leverage), Y-space residual distances, and Hotelling’s T² values | | | | |
| --- | --- | --- | --- | --- |
| **Observations (samples)** | **Predicted Responses**  **for Test and Training Data** | **Distance to X** | **Distance to Y** | **T² values** |
| F1 | 2.786815763 | 13179.48997 | 1.786815763 | 1.30161 |
| F2 | 2.465068126 | 18475.00168 | 1.465068126 | 1.79372 |
| F3 | 3.010697034 | 7677.367019 | 2.010697034 | 0.505751 |
| F4 | 3.150850562 | 11037.78841 | 2.150850562 | 0.31678 |
| F5 | 2.484863096 | 3393.207692 | 0.484863096 | 1.533528 |
| F6 | 1.733340941 | 25287.54575 | 0.266659059 | 4.03835 |
| F7 | 2.488152641 | 502.7187775 | 0.488152641 | 1.737655 |
| F8 | 3.112193888 | 22167.51572 | 1.112193888 | 1.627056 |
| F9 | 4.123151526 | 17964.25962 | 1.123151526 | 5.812939 |
| F10 | 3.148940661 | 4829.494318 | 0.148940661 | 3.362356 |
| F11 | 3.673061424 | 20296.93502 | 0.673061424 | 2.40351 |
| F12 | 3.845108092 | 17856.36458 | 0.845108092 | 0.740099 |
| F13 | 4.247375392 | 6687.069347 | 0.247375392 | 0.94621 |
| F14 | 3.683332952 | 6726.987831 | 0.316667048 | 1.582729 |
| F15 | 3.232868322 | 14743.51252 | 0.767131678 | 0.909981 |
| F16 | 4.925489193 | 31560.7299 | 0.925489193 | 4.328884 |
| F17 | 4.813611041 | 6456.138529 | 0.186388959 | 2.535228 |
| F18 | 4.623542869 | 6040.445557 | 0.376457131 | 1.667246 |
| F19 | 4.934091838 | 4748.464308 | 0.065908162 | 3.615776 |
| F20 | 4.827106061 | 2531.551553 | 0.172893939 | 3.846067 |
| F21 | 3.048123356 | 12855.52714 | 2.951876644 | 0.718931 |
| F22 | 3.253929222 | 1334.892598 | 2.746070778 | 0.080657 |
| F23 | 3.318148141 | 2582.235826 | 2.681851859 | 0.323551 |
| F24 | 3.070137858 | 1760.339092 | 2.929862142 | 0.271385 |
| Predicted responses were generated using the full dataset | | | | |

| **Table 15** Coefficients of the Partial Least Squares Discriminant Analysis (PLS-DA) model for both original and standardized input data | | |
| --- | --- | --- |
| Independent Variables | Coefficients for Original Data | Coefficients for Standardized Data |
| Intercept | 7.70794602 | --------------- |
| Pb | -0.022329278 | -0.27778 |
| Cu | 0.003640623 | 0.137493 |
| Zn | -0.013335267 | -0.22885 |
| Ni | -0.00635502 | -0.019 |
| Cr | 0.025259856 | 0.343845 |
| Mn | 1.87486E-05 | 0.059122 |
| Fe | -8.1837E-06 | -0.16888 |

**References**

Adimalla, N. (2020). Heavy metals contamination in urban surface soils of Medak province, India, and its risk assessment and spatial distribution. *Environmental Geochemistry and Health*, *42*(1), 59–75. https://doi.org/10.1007/s10653-019-00270-1

Al-Shidi, H. K., Sulaiman, H., Al-Reasi, H. A., Jamil, F., & Aslam, M. (2021). Human and ecological risk assessment of heavy metals in different particle sizes of road dust in Muscat, Oman. *Environmental Science and Pollution Research*, *28*(26), 33980–33993. https://doi.org/10.1007/s11356-020-09319-6

Cunha-Lopes, I., Alves, C. A., Casotti Rienda, I., Faria, T., Lucarelli, F., Querol, X., Amato, F., & Almeida, S. M. (2022). Characterisation of non-exhaust emissions from road traffic in Lisbon. *Atmospheric Environment*, *286*, 119221. https://doi.org/10.1016/j.atmosenv.2022.119221

Dat, N. D., Nguyen, V.-T., Vo, T.-D.-H., Bui, X.-T., Bui, M.-H., Nguyen, L. S. P., Nguyen, X.-C., Tran, A. T.-K., Nguyen, T.-T.-A., Ju, Y.-R., Huynh, T.-M.-T., Nguyen, D.-H., Bui, H.-N., & Lin, C. (2021). Contamination, source attribution, and potential health risks of heavy metals in street dust of a metropolitan area in Southern Vietnam. *Environmental Science and Pollution Research*, *28*(36), 50405–50419. https://doi.org/10.1007/s11356-021-14246-1

Ferreira-Baptista, L., & De Miguel, E. (2005). Geochemistry and risk assessment of street dust in Luanda, Angola: A tropical urban environment. *Atmospheric Environment*, *39*(25), 4501–4512. https://doi.org/10.1016/j.atmosenv.2005.03.026

Guo, W., Liu, X., Liu, Z., & Li, G. (2010). Pollution and Potential Ecological Risk Evaluation of Heavy Metals in the Sediments around Dongjiang Harbor, Tianjin. *Procedia Environmental Sciences*, *2*, 729–736. https://doi.org/10.1016/j.proenv.2010.10.084

Hakanson, L. (1980). An ecological risk index for aquatic pollution control.a sedimentological approach. *Water Research*, *14*(8), 975–1001. https://doi.org/10.1016/0043-1354(80)90143-8

Iwegbue, C. M. A., Nnanna, C. A., Ogwu, I. F., Odali, E. W., & Martincigh, B. S. (2024). Concentrations, sources and exposure to metals in dust from automobile mechanic workshops in Nigeria. *Journal of Trace Elements and Minerals*, *10*, 100186. https://doi.org/10.1016/j.jtemin.2024.100186

Malakootian, M., Mohammadi, A., Nasiri, A., Asadi, A. M. S., Conti, G. O., & Faraji, M. (2021). Spatial distribution and correlations among elements in smaller than 75 μm street dust: Ecological and probabilistic health risk assessment. *Environmental Geochemistry and Health*, *43*(1), 567–583. https://doi.org/10.1007/s10653-020-00694-0

Mostafa, M. T., Osman, M. S. M., Fahmy, W., Abu Elwafa, R. Y., El-Nady, H., Gomaa, R. M., Salman, S. A., & Khalifa, I. H. (2024). Geochemical characterizations, pollution monitoring, and health risks of toxic metals released from an active phosphate mine in the Western Desert, Egypt. *Journal of Sedimentary Environments*, *9*(4), 1013–1033. https://doi.org/10.1007/s43217-024-00206-5

Muller, G. (1969). Index of geoaccumulation in sediments of the Rhine River. *Geojournal*, *2*, 108–118.

Ren, M., Deng, Y., Ni, W., Su, J., Tong, Y., Han, X., Li, F., Wang, H., Zhao, F., Huang, X., & Huang, Z. (2024). Sources Analysis and Health Risk Assessment of Heavy Metals in Street Dust from Urban Core of Zhengzhou, China. *Sustainability*, *16*(17), 7604. https://doi.org/10.3390/su16177604

Tan, Z., Lu, S., Zhao, H., Kai, X., Jiaxian, P., Win, M. S., Yu, S., Yonemochi, S., & Wang, Q. (2018). Magnetic, geochemical characterization and health risk assessment of road dust in Xuanwei and Fuyuan, China. *Environmental Geochemistry and Health*, *40*(4), 1541–1555. https://doi.org/10.1007/s10653-018-0070-7

USEPA. (1996). *Soil Screening Guidance: User’s Guide*. U.S. Environmental Protection Agency: Office of Emergency and Remedial Response.

USEPA. (2002). *SUPPLEMENTAL GUIDANCE FOR DEVELOPING SOIL SCREENING LEVELS FOR SUPERFUND SITES*. U.S. Environmental Protection Agency: Office of Emergency and Remedial Response.

USEPA. (2005). *Guidelines for Carcinogen Risk Assessment*. U.S. Environmental Protection Agency, Risk Assessment Forum.

USEPA. (2011). *Exposure Factors Handbook*. U.S. Environmental Protection Agency.

USEPA. (2012). *EPA’s Integrated Risk Information System Program*. U.S. Environmental Protection Agency: Office of Research and Development.

Xu, S., Zheng, N., Liu, J., Wang, Y., & Chang, S. (2013). Geochemistry and health risk assessment of arsenic exposure to street dust in the zinc smelting district, Northeast China. *Environmental Geochemistry and Health*, *35*(1), 89–99. https://doi.org/10.1007/s10653-012-9463-1

Yesilkanat, C. M., & Kobya, Y. (2021). Spatial characteristics of ecological and health risks of toxic heavy metal pollution from road dust in the Black Sea coast of Turkey. *Geoderma Regional*, *25*, e00388. https://doi.org/10.1016/j.geodrs.2021.e00388

Yuan, D., Li, P., Yan, C., Wang, J., Bai, X., Wei, Y., Wang, C., & Kou, Y. (2025). Risk Assessment of Heavy Metals in Road-Deposited Sediments and Correlation Distribution of DOM and Heavy Metals in Beijing, China. *Toxics*, *13*(4), 308. https://doi.org/10.3390/toxics13040308
